# Predicted no effect concentrations of antifungals for wastewater management and agricultural use

**DOI:** 10.3389/ftox.2026.1767925

**Published:** 2026-03-24

**Authors:** D. Gil, S. José, A. Ascenso, M. Novak Babič, E. Segal, J. Meletiadis, J. P. Gangneux, C. J. Weiskerger, H. M. Solo-Gabriele, E. Valério, J. Brandão

**Affiliations:** 1 Department of Environmental Health, National Institute of Health Doutor Ricardo Jorge, Lisboa, Portugal; 2 Department of Biology, Biotechnical Faculty, University of Ljubljana, Ljubljana, Slovenia; 3 Department of Clinical Microbiology and Immunology, School of Medicine, Tel Aviv University, Tel Aviv, Israel; 4 Clinical Microbiology Laboratory, Attikon University Hospital, Medical School, National and Kapodistrian University of Athens, Athens, Greece; 5 Centre National de Référence des Mycoses et Antifongiques LA-AspC Aspergilloses chroniques, European Excellence Center for Medical Mycology (ECMM EC), Centre hospitalier Universitaire de Rennes, Rennes, France; 6 University Rennes, CHU Rennes, Inserm, EHESP, Irset (Institut de Recherche en Santé, Environnement et Travail), UMR_S 1085, Rennes, France; 7 United States Environmental Protection Agency, Atlanta, GA, United States; 8 Department of Chemical, Environmental, and Materials Engineering, University of Miami, Coral Gables, FL, United States; 9 cE3c – Centre for Ecology, Evolution and Environmental Changes, Faculdade de Ciências da Universidade de Lisboa, Lisboa, Portugal

**Keywords:** antifungal resistance, antifungals dispersion in environment, One Health, PNEC, systematic literature reviews, wastewater

## Abstract

Antifungal resistance is an on-growing public health concern due to the difficulty in managing or treating medical conditions that often favour fatal fungal infections. The changing climate and globalisation, which increase fungal persistence and propagation, adds to that concern. Wastewater disposal is one potential source to the environment as antifungals are released into it. Considering that most fungal infections originate from the environment and considering the One Health principle, introducing antifungals through wastewater effluents has the potential to promote the emergence and dissemination of antifungal resistance. The objective of this study was to generate knowledge that can assist regulating the release of antifungals in the environment by quantifying predicted no-effect concentrations (PNECs) that would not promote antifungal resistance. For this purpose, a systematic review was performed to consolidate information on antifungals released to the environment and respective concentrations. The systematic literature review followed Preferred Reporting Items for Systematic literature reviews and Meta Analyses extension for Scoping Reviews (PRISMA-SLR). The analysis of 122 reviewed articles using this approach showed high concentrations and dispersion of antifungals in water, wastewater or soil. This highlights their potential dispersion in the environment, thus increasing the potential of fungal antimicrobial resistance. Due to the lack of PNEC values using fungi as model organisms in this review, PNECs for 17 antifungals were calculated using *Candida albicans* as model, as it is done for clinical purposes. We consider that the antifungal PNECs calculated and consolidated from the literature can be used to prioritise them for regulation and to determine acceptable levels in wastewater effluents.

## 1 Introduction

### 1.1 Fungal contaminations

Microbial contamination in the environment is known to be a public health hazard. Faecal indicator bacteria (FIB) in recreational waters can cause a variety of health problems for swimmers and beach users (Bilbray, 2000), while the inadequate treatment of surface waters in preparation for drinking can lead to biological, chemical or even radiation related health issues for the public (Fawell and Nieuwenhuijsen, 2003; Havelaar and Melse, 2003; Li and Wu, 2019). While bacterial and chemical contaminants in waters have been monitored and managed for decades in countries with more developed economies, fungal contaminations still represent an emerging health concern (Zhao et al., 2022). Research has shown the prevalence of fungal contamination in swimming pools (Rezaei et al., 2021) as well as in water and sand at beaches (Brandão et al., 2021), even in drinking water (Kanzler et al., 2008; Pereira et al., 2009; Kadaifciler and Demirel, 2018) and wastewater (Viegas et al., 2014; Teixeira et al., 2020) in locations across the world. Despite these known fungal contamination concerns, management of fungi and policy related to public health effects is lacking.

### 1.2 Antifungal resistance

It is estimated that more than 300 million people worldwide are infected by fungi every year, and most of those infections are treated with antifungals of the azole class. The need for prolonged treatment and the ease of self-medication with azole antifungals contributes to the development of antifungal resistance (Manuel da S Azevedo et al., 2016).

While public health awareness, monitoring and management of fungal contaminants has lagged behind much of the research, another health hazard has emerged in recent years – antifungal resistance. Overall, antifungals are detected frequently and considered emerging contaminants in the environment (Assress et al., 2020). In addition to the toxicity they represent for living organisms, there is also the possibility that they may lead to the acquisition or transmission of resistance to existing medication (Santos et al., 2005). Resistance to antimicrobials in fungi is known to be promoted by fungal exposure to antifungals in the environment (Bottery et al., 2024). Resistance genes arise and propagate, both vertically and horizontally, sometimes across very unrelated species (Morogovsky et al., 2022; Bottery et al., 2024). Antimicrobials in the environment are thus highly undesirable as resistant microbes will thrive in their presence, resulting in the propagation of resistance genes making anti-microbials ineffective. This process has been witnessed with the emergence of resistance to azoles in *Aspergillus fumigatus* sensu stricto (Snelders et al., 2008; European Food Safety Authority et al., 2025; Sanseverino et al., 2025). The azoles used in agriculture are not chemically very different from those used in medical practice. The widespread use of these azoles has resulted in the emergence of resistance by this species, currently propagating throughout the world.

### 1.3 Ensuring public health safety

To effectively manage fungal contaminants and antifungal resistance in water, it is necessary to understand the risks that they pose to humans. Health outcomes, transmissibility, persistence in the environment and in a host can all depend on the species of fungi, but these life history characteristics are critical to the development of effective standards to ensure public health safety.

A important trait of chemical contaminants is their Predicted No-Effect Concentration (PNEC). This represents the highest concentration at which below no adverse effects of exposure in an ecosystem are measured. For antifungals this concept includes not promoting their resistance nor selection of resistant organisms. PNEC values are intended to be conservative and predict the concentration at which a chemical will not cause human health effects and can potentially reflect a safety threshold goal for water quality standards in relation to fungal contamination.

### 1.4 Wastewater quality assessment and the removal of antifungals

Wastewater receives antifungal agents as well as fungi that may be antifungal resistant. Conventional (and most common) wastewater treatment consists of two general categories: primary treatment, which focuses on the settling of wastewater and secondary treatment, which involves aeration of the wastewater. The secondary treatment process accelerates microbial consumption of dissolved organic matter and the removal of the microbial biomass through clarification (secondary settling), the efficacy of which is measured through decreases in biochemical oxygen demand (BOD) (Davis, 2020). Depending upon effluent requirements, wastewater may undergo more advanced treatment processes including tertiary treatment designed for the removal of nutrients such as nitrogen and phosphorous. At the end of the process, many communities employ disinfection of the wastewater to inactivate pathogenic microbes by the addition of an oxidant (e.g., chlorine or ozone) and/or through irradiation via UV light (Pepper et al., 2015). However, none of the conventional processes described are designed to remove trace organic pharmaceutical compounds, such as antifungals; therefore wastewater treatment generally does not completely remove antifungals resulting in the release of antifungal agents to the environment. Beyond fluconazole, other antifungals that persist through conventional wastewater treatment include bifonazole, naftifine, posaconazole, and ciclopirox (Bisceglia et al., 2010; Assress et al., 2019; Yang et al., 2022).

The efficiency of treatment process in removing these compounds in wastewater treatment plants reflects the conditions in partitioning of contaminants between water and solid phases. Key factors include water solubility, hydrophobicity [i.e., affinity to organic matter (Log(K_ow_)], and the dissociation constant (pKa), among others, which are summarized in Table 1.

**TABLE 1:** Basic information on some fungicides.

Antifungal	Structure	Formula	Cas number	Molecular weight (g/mol)	Water solubility (mg/L)	Log Kow	Log Koc	Log BCF	pKa	GHS classification
Carbendazim	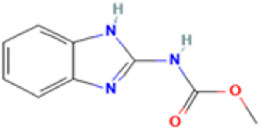	C9H9N3O2	10605-21-7	191.21	​	1.52	​	​	4.29	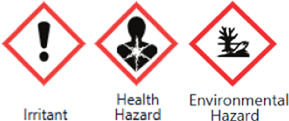
Climbazole	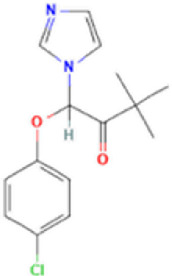	C15H17ClN2O2	38083-17-9	292.8	8.28	3.76	3.08	2.15	7.50	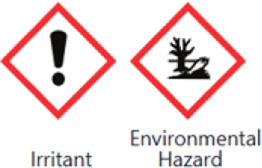
Clotrimazole	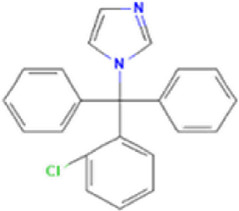	C22H17ClN2	23593-75-1	344.8	0.03	4.10	6.43	3.80	6.12	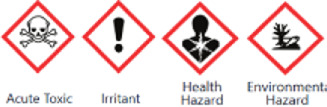
Econazole	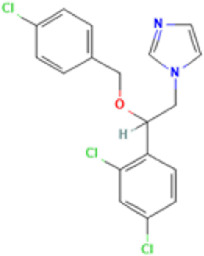	C18H15Cl3N2O	27220-47-9	381.7	1.48	5.61	5.53	​	6.77	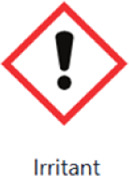
Fluconazole	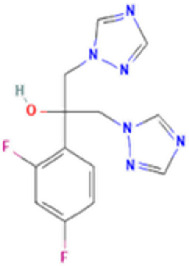	C13H12F2N6O	86386-73-4	306.3	1390	0.50	3.59	0.50	2.56	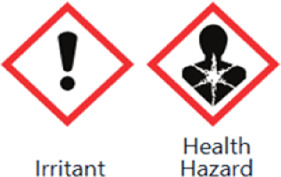
Griseofluvin	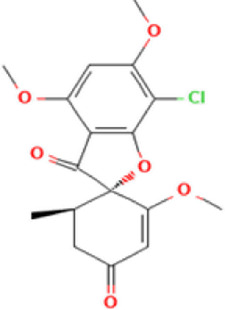	C17H17ClO6	126-07-8	352.8	8.64	2.18	​	​	​	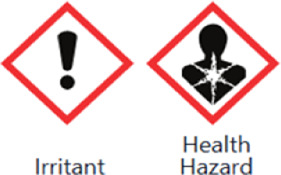
Imazalil	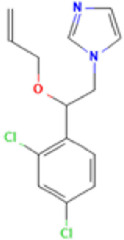	C14H14Cl2N2O	35554-44-0	297.2	​	3.82	​	​	​	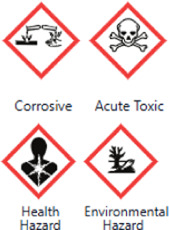
Itraconazole	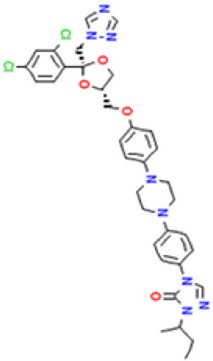	C35H38Cl2N8O4	84625-61-6	705.6	​	6.16	6.06	​	3.92	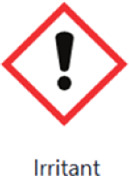
Ketoconazole	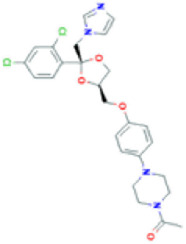	C26H28Cl2N4O4	65277-42-1	531.4	0.09	4.35	4.26	2.54	3.00	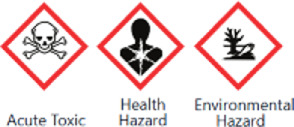
Metronidazole	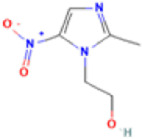	C6H9N3O3	443-48-1	171.15	11000	-0.02	​	​	2.38	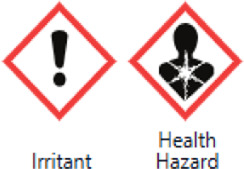
Miconazole	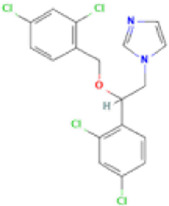	C18H14Cl4N2O	22916-47-8	416.1	0.01	6.25	5.74	3.79	6.65	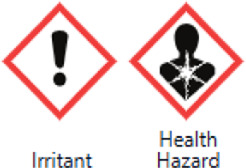
Posaconazole	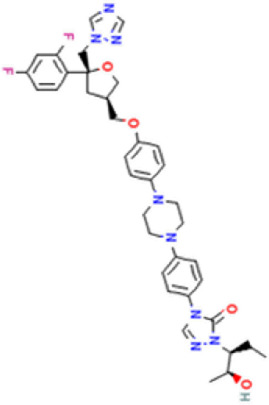	C37H42F2N8O4	171228-49-2	700.8	​	4.77	6.09	​	3.93	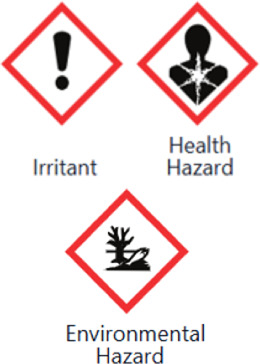
Propiconazole	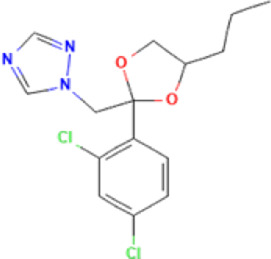	C15H17Cl2N3O2	60207-90-1	342.2	100	3.72	​	​	​	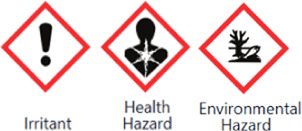
Tebuconazole	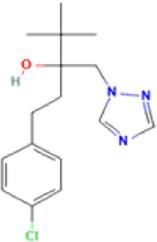	C16H22ClN3O	107534-96-3	307.8	36	3.7	2.85	​	5.0	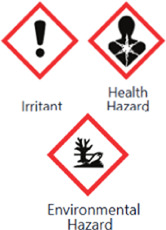
Terbinafine	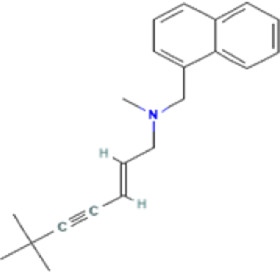	C21H25N	91161-71-6	291.4	0.738	5.9	​	​	7.10	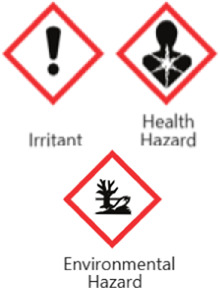
Thiabendazole	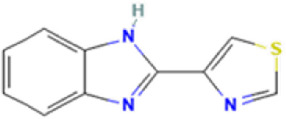	C10H7N3S	148-79-8	201.25	50	2.47	​	​	4.64	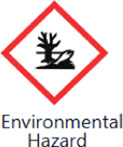
Voriconazole	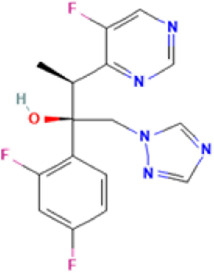	C16H14F3N5O	188416-29-7	349.3	​	1.57	3.692	​	2.27	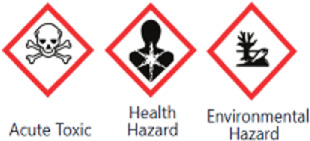

Total estimated biodegradation rate in WWTPs - (TEBR); Total estimated sludge adsorption in WWTPs - (TESAR); Total estimated removal rate in WWTPs - (TERR). Data retrieved from: Chen and Ying (2015); Assress et al. (2020); De Mello-Sampayo et al. (2024); Pubchem; PPDB: Pesticide Properties Database. Globally Harmonized System of Classification and Labelling of Chemicals (GHS).

Normally, compounds with a Log (K_ow_) >4 are lipophilic and are likely to be adsorbed in solid matrices (sludge, soil, sediment), so they will respond well to adsorption removal treatments. Compounds with Log (K_ow_) <2 tend to remain in the liquid phase of the treatment effluent. For hydrophilic-lipophilic compounds with values of 2 <Log (K_ow_) <4, it is possible that they could be in both the liquid and solid phases (Iancu et al., 2024).

## 2 Objective

The objective of this study was to generate knowledge to support future environmental regulations concerning the release of antifungals agents into the environment, particularly by addressing the Predicted No-Effect Concentrations (PNECs). To achive this, a systematic review was conducted in accordance with the Joanna Briggs Institute methodology (Joanna Briggs Institute, 2024) and reported following the Preferred Reporting Items for Systematic Reviews and Meta-Analyses (PRISMA 2020) guidelines (Page et al., 2021).

This review aimed to synthesize evidence regarding antifungal concentrations and their distribuition in environment matrices, including wastewater, rivers, surface water, groundwater, sludge, and sediments. Additionally, the study assessed whether PNEC values have been established for these compounds and whether such values could serve as reference points for future regulatory frameworks.

## 3 Materials and methods

This systematic review was conducted according to the Joanna Briggs Institute methodology (Joanna Briggs Institute, 2024) and reported following the PRISMA 2020 statement (Page et al., 2021), ensuring methodological rigor, transparency, and reproducibility. The completed PRISMA 2020 checklist is provided as Supplementary Material.

Objectives: Determine the antifungals’ concentrations and dispersion in the environment, as well as, addressing if there are PNEC values determined for these drugs that could be used as guidelines for future legislation.

### 3.1 Data source and search strategy

The methodology for conducting this systematic literature review adhered to the PRISMA (Preferred Reporting Items for Systematic Reviews and Meta-Analyses) guidelines (Page et al., 2021), using MeSH terms (Medical Subject Headings) (National Library of Medicine, 2024), as they are the standard for indexing health-related articles in databases.

Three major scientific databases: PubMed, Science Direct, and Web of Science, along with a Portuguese national scientific repository, RCAAP (Repositórios Científicos de Acesso Aberto de Portugal), were selected due to their relevance and comprehensiveness in science and health.

Initially, five pilot tests were conducted across the different databases. These tests involved entering search strings with multiple keywords (MeSH terms) into the databases. Based on the results, the search strings for this study were refined to ensure that some key articles about the theme were retrieved using the selected search strings, thus guaranteeing the sensitivity of our search strategy. In the case of RCAAP, due to language constraints (as searches were limited to Portuguese) and the platform’s functionality (which only returned results for queries containing a maximum of two terms, thereby limiting the comprehensiveness of the search), a total of 20 pilot tests were conducted.

On 7 February 2024, the above mentioned databases were systematically searched using the final determined search strings and strategic keywords relevant to the study’s focus:

In PubMed and Web of Science, we used search strings combining terms such as “wastewater AND (antifungal agents OR drug resistance, FUNGAL OR fluconazole OR itraconazole OR posaconazole OR voriconazole OR clotrimazole OR terbinafine OR anidulafungin OR caspofungin OR micafungin OR risk assessment)”.

For Science Direct, the search strings included “wastewater AND risk assessment AND (antifungal agents OR drug resistance, FUNGAL).”

In the RCAAP database, we used specific search terms in Portuguese: “águas residuais E antifúngicos” and “águas residuais E avaliação de risco.”

Our primary objective was to explore various categories of antifungals, notably azoles and echinocandins, within the context of different water types. We aimed to identify articles that addressed these antifungal classes in various water sources. Therefore, we used MeSH terms focused on antifungal resistance. These terms were strategically combined with logical operators ‘AND’ and ‘OR’ to construct comprehensive search queries, enabling us to capture a wide range of relevant literature. When simultaneous coverage was not feasible, our secondary objective was to find articles that independently addressed these antifungal categories, ensuring a thorough examination of antifungal resistance in diverse aquatic environments. These aquatic environments include wastewater, which is the focal subject of this investigation. Additionally, we also considered its corresponding influents and effluent streams within wastewater treatment plants, the sludge generated during treatment, sediments interfacing with these waters, and hospital effluents. Complementary to this category, we have also collected data sets for surface water, riverine systems, river basins, and groundwater reservoirs, since wastewater discharge may lead to their contamination with antifungal agents.

In addition to analysing the presence of antifungals in those different environments, the concentrations of these substances were also assessed.

### 3.2 Eligibility criteria

The initial search retrieved 13,527 records, including across the selected databases and registers, including scientific articles, books, and master’s theses.

The inclusion criteria were pre-defined to ensure a rigorous and transparent selection processed align with the study’s objetive. The publication period was restricted to records published between 2000 and 2024, reducing the total to 13,215 articles. This decision was based on the significant increase in fungal infections and multidrug resistance over the most recent decades, making it crucial to incorporate scientific data that reflects the contemporary global context. Additionally, the vast majority of the research conducted in this area has occurred since 2000, with 97.7% (13,215 out of 13,527) of all publications identified having been published during the 2000–2024 period, further supporting the chosen timeframe. After applying this criterion, the literature identified was uploaded in the Zotero, an open-source reference management system designed for storing and organizing bibliographic references.

To enhance transparency and reproducibility, exclusion criteria were established based on the PCC (Population, Concept, Context) framework:

Population: Studies that did not explicitly address antifungal agents or their environmental presence were excluded.

Concept: Articles mentioning antifungal agents but lacking quantitative data on their concentrations in aquatic environments or PNEC values were removed.

Context: Studies focusing solely on antifungal agents in laboratory or clinical settings, without any environmental relevance, were excluded.

Duplicate records were removed using Zotero’s built-in duplicate detection tool, leaving 9,837 unique results.

### 3.3 Data refinement and synthesis

A detailed manual refinement process was conducted to ensure the quality and relevance of the selected records. Indexes, dictionaries, conference abstracts, and documents containing only bibliographies lacking pertinent information were removed, resulting in a dataset of 9,640 articles.

The screening process was conducted in two main phases, Title and Abstract Screening and Access to Full-Text Records.

During the initial phase we meticulously examined article titles and abstracts to assess for their relevance to antifungal agents. Our validation process involved using previously established search terms, along with additional keywords such as “pharmaceuticals” and “contaminants” to capture studies indirectly mentioning antifungal compounds. Terms such “river,” “influent,” and “effluent” were included to identify studies addressing antifungal agents in wastewater discharge and aquatic environments.

### 3.4 Study risk of bias assessment

The initial screening was performed by two independent reviewers to ensure methodological rigor and minimize individual bias.

Discrepancies were resolved by a third reviewer, following the approach recommended by Wang et al. (2020), which highlights potential error rates in manual abstract screening.

Out of 685 potentially relevant articles, 70 were inaccessible due to institutional paywalls and the absence of available versions in open-access repositories, leaving 615 accessible articles. This high count of inaccessible records may introduce selection bias, as highlighted by Gartlehner et al. (2020), who emphasized the impact of limited access on evidence synthesis studies.

Among the accessible records (615), only studies published in English and Portuguese were considered (610).

**FIGURE 1:**
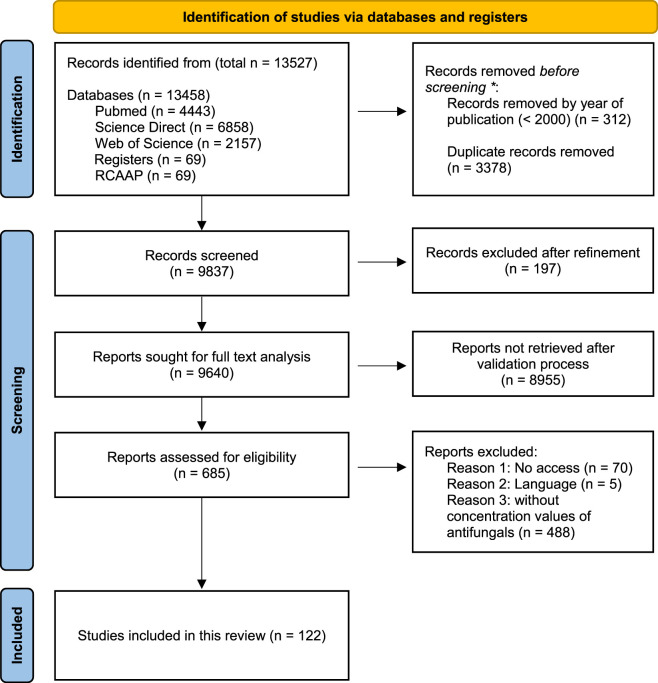
PRISMA 2020 flow diagram used for systematic literature review in this study, which included 244 searches of databases and registers only. * Using automation tool Zotero.

During full-text screening, articles that did not contain relevant antifungal data or that failed to provide quantitative concentration values of antifungal agents in aquatic environments (488) were excluded. After this refinement, a final set of 122 articles containing relevant antifungal concentration data was selected. Figure 1 presents the PRISMA 2020 flow diagram, illustrating the screening and selection process.

## 4 Results and discussion

### 4.1 Occurrence of antifungals in surface water, groundwater, wastewaters, and sludge

For simplification given the extension of the table, the results from the systematic literature review are presented in Supplementary Table S1, and the corresponding references are also within that file. This table shows the concentrations of antifungals across several geographical regions and various environmental matrices.

As a result of the systematic literature review carried out, we can observe that 54 different antifungals, mostly azoles, were found in either surface water, groundwater, wastewater, or sludge.

**FIGURE 2:**
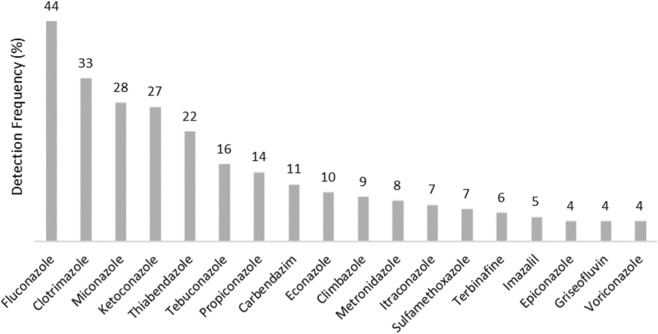
Detection frequency of antifungal compounds in environmental samples. The detection frequency (%) was calculated as the number of studies in which a given antifungal was reported divided by the total number of studies analyzed (122), multiplied by 100.

Of the 122 studies analysed, there were 371 mentions of antifungals detected in their samples, and it was possible to observe that azole antifungals were widely present in wastewater and sludge. These were persistent during treatment in the WWTPs, with varied fate depending on the compound. The frequency of detection of various antifungal compounds across 122 studies is represented in Figure 2. Fluconazole exhibited the highest detection frequency (44%), followed by clotrimazole (33%) and miconazole (28%). Other antifungals, such as voriconazole, griseofulvin, and epiconazole, showed lower detection frequencies (4%–5%). This distribution highlights the varying environmental presence of antifungal compounds, which may be influenced by their usage patterns, physicochemical properties, and environmental persistence.

From a scientometric perspective, the available literature reveals a pronounced geographical and temporal imbalance that likely reflects differences in data availability and research capacity rather than the real global distribution of the phenomenon under investigation. The majority of retrieved studies originate from Europe and Asia, with notable contributions from Spain (16 studies), Sweden (8 studies), Germany (6 studies), China (22 studies), and India (8 studies). The higher number of studies found in Europe, may be associated with the existence of monitoring programs for wastewater treatment plants and surface waters, alongside regulatory frameworks that encourage systematic data collection and reporting. In Asia, the recent increase in publications may be related to rapid urbanization, industrial expansion, and rising pharmaceutical use. In contrast, Africa and Latin America are substantially underrepresented, potentially reflecting limitations in monitoring infrastructure, research funding, and lack of visibility within international bibliographic databases or due to the publications not being in English. These disparities may introduce a possible source of bias in this review, as regions experiencing considerable environmental pressures could be insufficiently captured in the existing literature, but it is a snapshot of the data available.

From a temporal perspective, research activity spans the period from 2005 to 2023, displaying an increase in the number of publications after 2012. This trend may be associated with advances in analytical techniques and growing attention to emerging contaminants. The highest number of studies was observed in 2015, with 15 publications.

The compound described most frequently was fluconazole, in 54 reports, equivalent to 44% of the detection frequency (Figure 2), with a predominance in the aqueous phases. The highest concentration recorded was 236,950 μg/L in a sewer from an industrial area in India. Such a high value may reflect the particular conditions where it was detected, such as low water flow coupled with high temperatures, which could have resulted in the concentration of this contaminant in the sample (Lübbert et al., 2017). However, misuse and incorrect disposal can be major sources of contamination for the environment, with India and China providing large-scale production of these products for the whole world. The literature describes that the areas surrounding production zones are a major source of environmental contamination (Fick et al., 2009; Bengtsson-Palme and Larsson, 2016).

In another context, the author Lopez-Herguedas et al. (2022) found a maximum concentration of 109,480 ng/L for fluconazole. This occurrence was detected in an effluent from a water treatment plant located at the convergence of the Henares River and the Monjas stream (Spain), during the summer sampling period (June). The low affinity for organic matter of fluconazole (Log(K_ow_) = 0.50) (Table 1) may explain why it has rarely been detected in sludge and no results have been found in sediments (Lindberg et al., 2010; Peng et al., 2012). Peng et al. (2012) evaluated the permanence of the compounds in the various treatment phases, influent, effluent, and sludge, and fluconazole remained largely in the final effluent.

**FIGURE 3:**
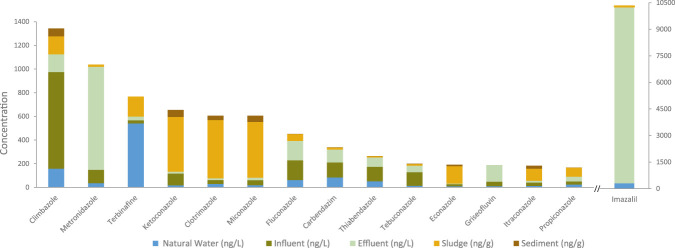
Median concentration of antifungals found in different environmental matrices retrieved by the systematic literature review performed.

It is important to note that the detection frequencies of antifungals in the environment retrieved from our study (Figure 2) should be interpreted with caution, as they are influenced by the analytical scope of individual studies, including the range of antifungals investigated. As a result, antifungals described as « frequently detected » may reflect higher research investment rather than environmental prevalence. This is exemplified by several poorly investigated compounds which were found, at least once, in high concentration in the systematic review, suggesting that substantial environmental release may not be adequately reflected. To give another perspective, we present in Figure 3 a summary of median concentrations of the most prevalent antifungals in water and soil matrices found in this study, acknowledging the limitations discussed above. Climbazole, metronidazole, and terbinafine were among the compounds with the highest median concentrations, while imazalil exhibited an exceptionally high concentration in effluent (19,802 ng/L) (Supplementary Table S1 for details), requiring a break in the scale for proper visualization. These concentration levels are mostly related to the hydrophilic or lipophilic properties of each compound, as previously discussed.

If we take a closer look at the compounds with the highest median concentrations, climbazole was found in only 11 studies but detected in a concentration of 1,350 ng/L by Wick et al., (2010); in a municipal wastewater in Germany. This compound is an integral part of many everyday products that are widely and frequently used, such as shampoos, body creams, shower gels and specific products for hair loss and dandruff problems. Metronidazole (an antibiotic used to treat skin infections, *Clostridium difficile* colitis and bacterial vaginosis) was founded in only 10 studies, however Faleye et al. (2019) reported a high concentration of approximately 164,264 ng/L in an influent, as a result of a study carried out in wastewater treatment plants, based on the incidence of different diseases in a region of South Africa. One would expect this very high concentration to alarm researchers and water managers alike, leading to additional studies. However, this does not yet appear to be the case. Similarly, terbinafine, which was mentioned in only 7 occurrence records, has been detected in surface waters in India, in a lake at a concentration of 15,000 ng/L (Fick et al., 2009).

Other azoles, such as ketoconazole, itraconazole, miconazole, clotrimazole, econazole (Assress et al., 2019), and posaconazole (Martin and Hart, 2023), exhibit higher Log(Kow) values and low affinity for aqueous phase due to their lipophilic properties (Table 1), leading to their accumulation in biosolids. Similarly, the allylamine antifungal terbinafine shows comparable properties and accumulates in biosolids (Östman et al., 2017). Compounds reported in sludge, include clotrimazole, with a maximum concentration of 2,547 ng/g, detected in February in Guangzhou, China (Peng et al., 2012); ketoconazole found in 10,000 ng/g concentration by Östman et al. (2017) in a Swedish WWTP; and miconazole reports presence in sludge of 2,069 ng/g (Castro et al., 2021). The occurrence of these compounds in sludge has been demonstrated in several studies (Lindberg et al., 2010; Chen et al., 2012), but not exclusively; there are also records of occurrences in influent of wastewater. The authors J. Van De Steene and Lambert (2008) reported the occurrence in influent of United Kingdom for ketoconazole (143,000 ng/L) and miconazole (15,000 ng/L), showing that these compounds reach the WWTPs in high concentrations.

Other antifungals - including isoconazole, thiabendazole, sertaconazole, fenticonazole (Castro et al., 2016; 2021), climbazole, epoxiconazole, tebuconazole, imazalil, metconazole, and procloraz (Iancu et al., 2024) have also been detected at elevated concentrations in biosolids. The removal efficiency of climbazole was reported as <80%. Notably, clotrimazole, ketoconazole, and miconazole were almost entirely removed from the aqueous phase (effluent), demonstrating their strong affinity for hydrophobic particles and subsequent adsorption onto sludge. Studies have reported high concentrations of these antifungals in sludge, with values reaching 2,547 ng/L, 1800 ng/L, and 2069 ng/L, respectively (Lindberg et al., 2010; Chen et al., 2012; Peng et al., 2012).

Taking the above-mentioned information into account we must highlight that the practice of using sludge as a soil fertilizer represents a direct route for these contaminants to enter the soil. It also constitutes a long-term soil exposure problem. There is currently no legislation on the acceptable levels of antifungals during the application of sludge to soils, despite the new urban wastewater European directive 2024/3019, articles 20 and 21, mention sludge managements and monitoring.

Due to the lack of specific unit operation for their extraction, the treatment of antifungals at conventional wastewater treatment plants has been inconsistent (Monapathi et al., 2021; Wroński et al., 2024). For hydrophilic compounds, it is necessary to develop more efficient removal methodologies. In the case of lipophilic compounds, sludge adsorption makes a major contribution to the process of removing these compounds from the aqueous phase (Chen and Ying, 2015). The inconsistency in the elimination of antifungals from wastewater and/or their accumulation in biosolids, requires new innovative technologies to target a more efficient removal of the antifungals. Promising technologies include membrane biological reactors (MBR) which enhance treatment through membrane filtration as part of processing the effluent from secondary treatment. A study in South Africa (Holton et al., 2022) evaluated both antifungals (metronidazole and ketoconazole) and antifungal metabolites (metronidazole hydroxy and ketoconazole N-deacetyl) before and after treatment of wastewater incorporating MBR technology. Results showed a 60% to near 100% removal of metronidazole and a 40%–75% removal of its metabolite. For ketoconazole, removals were in the 60% to near 100% range, with no detection of its metabolite in the effluent. The relatively high removal rates at the wastewater treatment plants were attributed to the inclusion of an MBR, which provided ultrafiltration capacity for the wastewater. Due to the relatively small pore size of ultrafiltration membranes (0.2–0.5 μm), the MBR process enhanced the reductions of antifungals that have a propensity to partition to the particulate phase. Additional promising technologies for the removal of antifungal compounds include less common wastewater treatment processes such as novel sorbents such as hydrogels (Kumari et al., 2024) and graphene (Kogut et al., 2022). Electrochemical advanced oxidation (Lozano et al., 2022; de Souza et al., 2023), and photooxidation (Brice et al., 2022) have also been shown to reduce antifungals. Photocatalysis processes may also be considered. Photocatalysis includes the use of innovative nanomaterials (Gadore et al., 2024) that are activated through different forms of light such as low pressure fixed UV (256 nm) (Demissie et al., 2023), broad spectrum pulsed light (200–1,100 nm), and blue light (400–500 nm) (Garvey et al., 2022).

Regarding the fungicide tebuconazole, according to the studies analysed in this review, it was mostly detected in surface water. However, Stamatis et al. (2010) determined its highest concentration in wastewater (1,893 ng/L). Higher concentrations of tebuconazole were observed in WWTPs where rainwater and wastewater of domestic and industrial origin were treated. These results indicate that leaching can occur from parts of buildings treated with paints or coatings containing fungicide products, which then end up in the sewer as surface runoff (Stamatis et al., 2010).

In surface waters, the occurrence of fungicides from urban areas is often continuous throughout the year and the emission does not follow a seasonal pattern. This idea is supported by the fact that throughout the winter months, when fungicides are less commonly used in agriculture, the concentration detected in urban areas remains relatively constant. Carbendazim occurrences in WWTP samples have also been related to leaching due its use as a film-preservative in paints, coatings and roof sealings, as reported in Wick et al. (2010), with a maximum concentration in an influent of 143.0 ng/L.

Agricultural practices are the biggest source of pesticide contamination; such is the case of thiabendazole with the quantification of 120,000 ng/L in an effluent from a treatment plant located near a rural area in Spain (Romero-Cano et al., 2015). However, the processes carried out in the food industry may also be a major source of contamination that has not yet been widely studied. The fungicides imazalil (19,802 ng/L), and thiabendazole (926 ng/L) were found in an effluent from a food industry wastewater, values much higher than those found in an urban effluent in which imazalil (496 ng/L), and thiabendazole were not found (Campos-Mañas et al., 2019). The maximum concentration of thiabendazole detected in surface water was 34,500 ng/L in the River Mijares in Spain. The author linked this to the frequent use of this fungicide in the post-harvest processing of citrus fruits, which eventually leads to the discharge of contaminated effluents from the treatment plants that process the wastewater from these industries (Bijlsma et al., 2021). Also, in the Castelló area, Romero-Cano et al. (2015) detected a concentration of 1,900,000 ng/L in the influent of a WWTP.

In an environmental context, there are several records of the presence of antifungals in wastewater, surface water, sludge, and sediment in various countries such as Germany, Spain, Sweden, China, Switzerland, UK, Belgium, Canada, and Romania (Huang et al., 2010; Peng et al., 2012; Chen and Ying, 2015; Assress et al., 2019; 2020; Pathadka et al., 2022; Iancu et al., 2024). The use of azoles in agriculture is also widely known and taking place constantly (Brauer et al., 2019). Between 2010 and 2012, about 120,000 tons of azoles were sold, of which 119,000 were plant protection products (European Food Safety Authority et al., 2025).

The use of azole antifungal compounds is not only for pharmaceutical purposes through oral or topical prescriptions. There is also a widespread and frequent use of these compounds in everyday products such as shampoos, body creams, soaps, toothpaste, shower gels and specific products for hair loss, which specifically contain the antifungals ketoconazole and climbazole (Peng et al., 2012). As result of the frequent use of these compounds, their flushing in greater quantities into wastewater treatment systems is significant. Additional, substantial sources of emission of these antifungals are municipal and hospital wastewater.

In wastewaters, fungi are exposed directly to antifungals, adding selective pressure to antifungal resistance and, if viable in the effluent or sludge, they will be released into the environment along with any antifungals that remain in the water effluent after the wastewater treatment process (World Health Organization, 2022).

We must stress that the high concentrations of these antifungals in natural waters, effluents that will be released into the environment (Assress et al., 2020), or sludge that may be repurpose has fertilizer, raises a significant concern, especially if we consider the One Health approach.

Outside the pharmaceutical industry, antifungals have never been in-scope for wastewater regulation for surveillance or treatment in some countries like the United States of America (U.S. Environmental Protection Agency, 2024a). This means that their introduction in the environment by wastewater treatment systems is not currently monitored. The recently published European directive 2024/3019 of November 2024 does not contemplate antifungal agents either, despite them being addressed in the GLOWACON initiative (https://wastewater-observatory.jrc.ec.europa.eu/#/content/glowacon). Given the lack of regulation, monitoring, and limited awareness surrounding antifungals, questions remain about their dispersal in the environment and the consequences of lack of action.

Controlling the propagation of antimicrobials through wastewaters is one of the possible actions to fight resistance in both microbes and fungi. Wastewaters should never be reintroduced in the environment without proper treatment and removal of chemicals of relevance, such as antimicrobials and endocrine disruptors (Rodríguez-Hernández et al., 2022). Monitoring studies have revealed the presence of pharmaceutical products in the effluent from wastewater treatment plants, which shows that conventional treatments are not able to remove all the contaminants what could pose a potential risk to public health (Assress et al., 2020; Iancu et al., 2024).

The fact that there is no legislation imposing legal limits on the presence of these compounds has led the European Union to take preventive and monitoring measures in this area. To this end, a set of rules was implemented to protect surface- and groundwater bodies from deterioration, known as the “Watch List”; which defines the contaminants to be monitored. Over the years, this directive has been updated, with the first document appearing in 2015 and revised in 2022 (published as the implementing decision 2022/1,307 (European Commission, 2022). Very recently, the European Commission’s Implementing Decision (EU) 2025/439 of 28 February 2025 presents the most up-to-date Watch List of Substances for Union-wide monitoring in the field of water. This last list includes, among other compounds, antifungal azoles (clotrimazole, fluconazole, and miconazole) and pesticides azoles (Bromuconazole, Climbazole, Ciazofamide, Difenoconazole, Epoxiconazole, Itraconazole, Cetoconazole, Mefentrifluconazole, Propiconazole, Triticonazole) (European Commission, 2025).

Similarly, the United States of America does not currently implement water quality standards at the national levels for antifungal contaminants in surface water bodies or point source effluents such as wastewater treatment plants. At the state level, some azole antifungals used in agricultural settings are managed, such as difenoconazole, propiconazole and tebuconazole in the state of Montana (U.S. Environmental Protection Agency, n. d.). However, in October of 2024, the United States Environmental Protection Agency (USEPA) finalized a framework for collaboration with the US Department of Health and Human Services (HHS) and the United States Department of Agriculture (USDA) to evaluate and assess the propensity of antifungal use to contribute to antifungal resistance and public health hazards (U.S. Environmental Protection Agency, 2024b). The framework is specifically aimed at using a One Health approach to balance the need to antifungal compounds for medical treatment with the recognition that their discharge to the environment can promote antifungal resistance. The One Health approach, recognizing the impacts of antifungals in their use and disposal, could potentially open the door to further research and regulation of antifungal compounds in the environment, to protect human and environmental health.

### 4.2 The MIC approach for the determination of PNECs

There is little information available in the literature on the Predicted No Effect Concentration (PNEC) values of these antifungal compounds. The studies analysed in the systematic literature review performed present 14 PNEC values using models such as vertebrates, algae, crustaceans and bacteria (Supplementary Table S1). However, many drugs do not have environmental toxicity data, and therefore the PNECs cannot be determined. Yet, there is a need to define PNECs for those drugs in order to quantify and minimize the potential for the development of resistance. One empirical approach is to determine the PNECs based on the Minimal Inhibitory Concentrations (MIC). The MIC is the lowest drug concentration that substantially (>50%) inhibits fungal growth after 24 h for yeasts and 48–72 h for moulds. According to the methodology in Bengtsson-Palme and Larsson (2016), the 1% of the lowest observed MICs were determined for each antibiotic (MIC_1%_). The PNEC-MIC was then calculated as MIC_1%_/10 to account for the differences between MICs and the lowest concentration of an antibiotic that results in selection of resistance and competitive advantage based on growth rate (Andersson and Hughes, 2014). Although no significant changes in cell fitness and aggregation formation were observed during the exposure of *C. albicans* with sub-MIC caspofungin, a decrease in its respiratory metabolism and an increase in persister cells was found (Ye et al., 2022). Sub-MIC effects have also been described for caspofungin and *Aspergillus fumigatus* that may contribute to tolerance (Walker et al., 2015). Sub-MIC effects (growth reduction) in *Aspergillus* spp. were observed at 2-4 two-fold dilutions lower than the respective MIC for voriconazole (0.06 vs. 0.25–1 mg/L); although for another experimental antifungal drug, olorofim, similar sub-MIC effects were found for 23 two-fold dilutions below the MIC (Kühbacher et al., 2024). There is also *in vivo* evidence that the sub-MIC effects of fluconazole contribute to the selection of *C. albicans* resistant mutants in animal models (Andes et al., 2006).

To estimate PNEC-MIC for the antifungals we report in this study, we analysed the MIC distribution of *Candida albicans* to have the most conservative and robust PNEC-MIC, as *C. albicans* was the most susceptible species and the species with most MIC data on the EUCAST website (EUCAST, 2023) (www.eucast.org). Despite environmental fungi differing in ecological and resistance dynamics, the evidence available indicates that they generally exhibit equal or higher MIC values compared to *C. albicans* (www.eucast.org). For drugs that have no MIC distribution on the EUCAST website, we used information from previously published studies. MIC distributions were analysed with non-linear regression analysis using the Normal distribution model and the MIC corresponding to <1% of the MICs were estimated. The PNEC-MIC was thus defined by dividing MIC_1%_ by 10 (Table 2), following the methodology of Bengtsson-Palme and Larsson (2016). These values may serve as a starting point to determine environmental PNECs, based on experimental data.

**TABLE 2:** Predicted no-effect concentrations based on minimal inhibitory concentration of antifungal drugs.

Drug	Modal MIC (mg/L)	Observed lowest MIC (mg/L)	R^2^ of normal distribution fit	MIC_1%_ (mg/L)	PNEC-MIC (ng/L)
Amphotericin B	0.25	0.008	0.976	0.016	1,600
Flucytosine	0.25	0.06	0.986	0.032	3,200
Micafungin	0.008	≤0.004	0.994	0.0005	50
Anidulafungin	0.004	≤0.002	0.998	0.0003	30
Caspofungin^a^	0.5	0.06	0.969	0.063	6,300
Terbinafine^b^	0.008	≤0.008	0.724	0.001	100
Rezafungin	0.002	≤0.002	0.999	0.001	100
Fluconazole	0.25	0.03	0.999	0.032	3200
Voriconazole	0.016	≤0.002	0.994	0.002	200
Posaconazole	0.016	≤0.008	0.954	0.004	400
Itraconazole	0.016	≤0.016	0.983	0.002	200
Isavuconazole	0.004	≤0.002	0.999	0.001	100
Miconazole^c^	0.016	≤0.004	0.933	0.002	200
Econazole^c^	0.016	0.008	0.970	0.002	200
Clotrimazole^c^	0.008	≤0.004	0.995	0.002	200
Ketoconazole^c^	0.008	≤0.004	0.986	0.002	200
Fenticonazole^c^	0.5	0.125	0.986	0.063	6300

^a^ Unpublished data.

^b^ Arendrup et al. (2020).

^c^ Kroustali et al. (2025).

If we compare the PNEC values (ng/L) calculated via the MIC approach (Table 2) with those retrieved from the systematic literature review (Supplementary Table S1), we observe three antifungals used for clinical purposes on both approaches, clotrimazole, fluconazole and ketoconazole, and they present higher PNEC values generated by the PNEC-MIC approach (Table 3). We must enphasize that the model organisms used by these two approaches are quite different and probably, so are the effects of the antifungals on them (toxicity and/or resistance). Since the focus of this paper is to address the dispersion of antifungals in the environment and their potential implication in the promotion and selection of antifungal resistance of fungi, we consider the *Candida albicans* antifungal resistance model as more compatible with the objectives of this study, given its clinical relevance.

**TABLE 3:** Comparison of the PNECs values of antifungals obtained by the systematic literature review with the ones using the MIC approach using *C. albicans* as model. Data from Supplementary Table S1 - Systematic literature review.

Antifungal	PNEC (ng/L)	Model organism(s) used	PNEC-MIC (ng/L) for *C. albicans*
Azoxystrobin	200	-	-
Benzotriazole	97,000	Algae, plant, fish	-
Carbendazim	21,7	Crustaceans	-
Climbazole	560	Algae, plant, fish, crustaceans	-
Clotrimazole	<100	Bacteria, algae, plant, crustaceans, vertebrates	200
Ethylparaben	23,000	-	-
Fluconazole	613	Algae, fish	3200
Ketoconazole	50	Algae, fish, crustaceans	200
Methylparaben	15,000	​	-
Metronidazole	125	-	-
Propiconazole	10	-	-
Sulfamethoxazole	16,000	-	-
Triclocarban	661	-	-
Triticonazole	2,5	Crustaceans	-

We observe that several studies have found concentrations above the PNEC value presented in Table 2, calculated on the basis of the minimal inhibitory concentrations (MICs) using *Candida albicans* as a model. In particular for fluconazole the PNEC was found to be greater than 3,200 ng/L in the studies of Lübbert et al. (2017), Westlund and Yargeau (2017), Assress et al. (2020) and Lopez-Herguedas et al. (2022); voriconazole >200 ng/L (Lübbert et al., 2017); itraconazole >200 ng/L (Castro et al., 2016); miconazole >200 ng/L (Van De Steene et al., 2010; Peng et al., 2012; Castro et al., 2016; Velpandian et al., 2018); clotrimazole >200 ng/L (Peng et al., 2012; Yang et al., 2022); ketoconazole >200 ng/L (Östman et al., 2017; Dehm et al., 2021; Burzio et al., 2022; Holton et al., 2022) and terbinafine >100 ng/L (Fick et al., 2009; Bisceglia et al., 2010; Östman et al., 2017; Velpandian et al., 2018). The compounds econazole and fenticonazole were found in concentrations below the PNEC value. For the other compounds for which the PNEC value was calculated, no occurrence results were found in the records of the systematic literature review performed.

Because imazalil isn’t routinely tested against *C. albicans*, it can be estimated for example, either using target-enzyme potency as a proxy (IC_50_ for *C. albicans* CYP51 ≈ 0.082 µM ≈ 24 μg/L) giving a PNEC of ≈0.024 μg/L following Trösken et al. (2006) or assuming *Candida*-like MICs similar to potent imidazoles/dermatophyte data using a MIC 0.03–1 μg/mL = 30–1,000 μg/L, which results in a PNEC between 0.03 and 1 μg/L following (Galuppi et al., 2010). As mentioned afore, this compound has been reported at a concentration of 19,802 ng/L (Campos-Mañas et al., 2019).

## 5 Conclusion

As a result of the frequent use of the azole compounds without retention during wastewater treatment, they are disposed in high quantities into wastewater treatment systems. The main sources of these contaminants are urban and hospital wastewater. In the case of orally administered antifungals, they are only partially metabolised in the human body, and the rest is eliminated from the body unaltered into the sewage system. However, the use of azole antifungal compounds is not only happening in a pharmaceutical context. These compounds are an integral part of countless everyday products that are more widely and frequently used, such as shampoos, body creams, soaps, toothpaste, shower gels and specific products for hair loss problems that specifically contain the antifungals ketoconazole and climbazole (Peng et al., 2012). In agricultural contexts, the most common antifungal agent detected is the fungicide tebuconazole (Stamatis et al., 2010; Casado et al., 2019; Wroński et al., 2024).

The combination of the dispersion of environmental *Aspergillus* clones selected under the pressure of fungicides used in agriculture and farming, followed by drug pressure after long-term treatments of chronically infected patients, account sinergetically to the recent emergence of resistance to antifungals (Guegan et al., 2021). Also, some species of *Candida* spp. have intrinsic or emerging resistance to azoles (Whaley et al., 2017). The pressure of the presence of these compounds in the environment causes changes in mycological communities by favouring species which are resistant; this may lead to shifts of the prevalence of different species of *Candida* spp. in clinical cases. Additionally, the very strong association of dematiaceous fungi with materials and water in urban settings (Novak Babič et al., 2022) raises concerns of emergence of AMR in this particular group of fungi. In general, the emergence of antimicrobial resistance by fungi is very much an adaptation to our behaviour and needs to be controlled.

The systematic literature review here performed highlights the widespread presence of antifungals, especially azoles in various environmental matrices, including surface water, groundwater, wastewaters (influent and effluent), and sludge. Despite significant research on compounds like fluconazole, which are frequently detected, other antifungals such as climbazole, metronidazole, and terbinafine were found at very high concentrations, despite limited research found about these compounds being released in WWTP effluents. Their persistence in wastewater treatment plants and their eventual release into the environment, especially through sludge used as soil fertilizer, raises concerns about long-term exposure and potential ecological impacts. The study emphasizes the need for more comprehensive monitoring and regulation, as well as a better understanding of the environmental fate of these chemicals to mitigate their potential risks.

The determination of PNECs for antifungal compounds is a challenge due to the limited availability of environmental toxicity data. By applying the MIC-based approach, we estimated PNEC-MIC values as a conservative proxy to minimize the potential for resistance selection.

### 5.1 Recommendations

There is an urgent need for a One Health approach, integrating environmental, human, and animal health perspectives to address antifungal pollution. Without coordinated global efforts to monitor and regulate these contaminants, the risk of widespread antifungal resistance may increase, threatening the efficacy of life-saving antifungal treatments. Support and interagency collaboration frameworks are needed for identifying antifungal resistance sources and developing water quality standards under regulations such as the United States Federal Insecticide, Fungicide, and Rodenticide Act (FIFRA), WHO guidelines on wastewater, the EU’s recently published Urban Wastewater Directive, and so many others. Our findings also emphasize the need for improved wastewater treatment technologies, including advanced oxidation processes, membrane bioreactors, and other innovative methods that specifically target antifungals.

In short, the employment of calculated PNECs as a reference in enforced regulation can help delay/reduce the emergence azole resistance and this is currently non happening outside wastewater from specific segments of the industry. Stricter regulations on antifungal disposal and agricultural application should be considered to mitigate environmental contamination and resistance development, considering the PNEC values presented/determined in this study (Table 2).

### 5.2 Lessons for the future

The release of free azoles in the environment seems to be generally accepted as the cause of the emergence of azole-resistance in *Aspergillus fumigatus* sensu stricto.

The fate of echinocandines in wastewater is unreported in scientific literature thus far. Nevertheless, echinocandines are released to the environment because wastewater is not usually adequately treated for antifungals. Therefore, we anticipate that there will eventually be an emergence of resistance to the echinocandines class of antifungals from the environment. Given the properties of this antifungal we suspect that free molecules will stay dissolved in the treated water (caspofungin and anidulafungin) or attach to the sludge (micafungin and rezafungin), depending on their hydrophilia. Treatment of echinocandines may also be possible through Advanced Oxidation Processes (AOPs), which include the use of strong oxidants (e.g., ozone, hydrogen peroxide, or UV light) to break down complex molecules. Also, for treatment, it is possible that the hydrophilic echinocandines (caspofungin and anidulafungin) can be adsorbed by activated carbon.

The conclusions retrieved from this review could be relevant for stakeholders in other regions of the world. The methodological approaches, analytical frameworks, and monitoring strategies identified in this literature can serve as transferable references for regions where systematic data collection is still limited. In particular, regulators and water management authorities in underrepresented regions may use these findings to revise their existent monitoring programs (or implementation in cases where they lack), support to choose the target compounds, and anticipate potential environmental risks associated with emerging contaminants. Moreover, the identified knowledge gaps highlight opportunities for capacity building and international collaboration.
