# Supplementary material for: Predicted no effect concentrations of antifungals for wastewater management and agricultural use
Source: Front Toxicol. 2026 Mar 24;8:1767925. doi: 10.3389/ftox.2026.1767925 (PMC13055602; doi:10.3389/ftox.2026.1767925)
Supplement: Supplementary file 1 [file Table1.docx]

**Supplementary Material Gil et al.**

**Table S1:** Summary table retrieved from the Scoping Reviews (PRISMA-ScR) identifying the antifungal concentration levels and PNEC values in water, wastewaters and sediments.

| **Antifungals** | **PNEC (ng/L)** | **Concentration** | | | | | **Location** | **Reference** |
| --- | --- | --- | --- | --- | --- | --- | --- | --- |
|  |  | **Water (surface (river, dam), ground and drinking) (ng/L)** | **Wastewater** | | | **Sediment (ng/g)** |  |  |
|  |  |  | **Influent (raw) (ng/L)** | **Effluent (ng/L)** | **Sludge (ng/g)** |  |  |  |
| Albendazole | - |  | 763610.9 **(a)** |  | 555.4 |  | South Africa | Faleye *et al.*, 2019 |
| Azoxystrobin | 200.0  (River) |  |  |  |  |  | Korea | Lee *et al.*, 2024 |
| Azoxystrobin | - | 0.0  (Watershed) |  |  |  |  | Cameroon | Branchet *et al.*, 2018 |
| Azoxystrobin | - | 2.5 - 45.2 **(b)**  (River) |  |  |  |  | China | Peng *et al.*, 2018 |
| Benzothiazoles | - |  | 116000.0 | 43400.0 | 85700.0 |  | India | Karthikraj and Kannan, 2017 |
| Benzotriazole | 97000.0 | 1.4 - 1732.0 **(b)**  (Riverine) |  |  |  | 0.1 - 160.0 **(b)** | China | Zhang *et al.*, 2015 |
| Benzotriazole | - |  | 1200.0 **(a)** | 560.0 **(a)** |  |  | Sweden | Östman *et al.*, 2019 |
| Benzylparaben | - | <0.2 – 4.4 **(b)**  (River) | <0.2 – 4.1 **(b)** | 0.2 – 16.0 **(b)**  (Secondary Treatment) |  |  | Switzerland | Jonkers *et al.*, 2009 |
| Bifonazole | - |  | 0.1 - 23.8 **(b)** | <LOD – 15.7 **(b)** |  |  | China | Yang *et al.*, 2022 |
| Butoconazole | - | 1.5 ± 0.3 **(a)** | 1.6 ± 0.2 **(a)** | ND |  |  | China | Wang *et al.*, 2018 |
| Butylparaben | - | <0.2 – 2.8 **(b)**  (River) | 9.7 – 864.0 **(b)** | <0.2 – 12.0 **(b)**  (Secondary Treatment) |  |  | Switzerland | Jonkers *et al.*, 2009 |
| Carbendazim | - | <0.05 – 118.0 **(b)**  (River) |  |  |  |  | Spain | Rico *et al.*, 2019 |
| Carbendazim | - | 0.3 - 213.9 **(b)**  **(**Rampura canal, Aftabnagar lake, and Dasherkandi Trimohoni**)** |  |  |  |  | Bangladesh | Angeles *et al.*, 2020 |
| Carbendazim | - | 5.5 / 49.9 **(b)** (Watershed) |  |  |  |  | Cameroon | Branchet *et al.*, 2018 |
| Carbendazim | - | 1.4 - 146.0 **(b)** (river) |  |  |  |  | China | Peng *et al.*, 2018 |
| Carbendazim | 21.7 | 18.0 **(d)**  (Xizhijiang River)  48.8 **(d)**  (Shima River) |  |  |  | 3.4 **(d)**  (Xizhijiang River)  3.5 **(d)**  (Shima River) | China | Chen *et al.*, 2014 |
| Carbendazim | - | 11.0 – 370.0 **(b)** |  | 25.0 – 210.0 **(b)** |  |  | China, USA, Philippines and India | Angeles *et al.*, 2021 |
| Carbendazim | - | 6.9 ± 0.3 **(d)**  (River Reservoir) | 110.0 ± 8.0 **(d)** | 114.0 ± 4.0 **(d)** | 34.6 ± 1.7 **(d)**  (Dewatered) | 1.4±0.2 **(d)**  (Soil) | China | Chen *et al.*, 2012 |
| Carbendazim | - |  | 7.5 - 97.1 **(b)** * | 2.3 - 21.1 **(b)** * | 0.05 - 3.2 **(b)** * |  | China | Liu *et al.*, 2017 |
| Carbendazim | - | 18.0 ± 1  (River)  94.0 ± 22  (Stream 1)  84.0 ± 4  (Stream 2) | 41.0 ± 6  (WWTP 1)  143.0 ± 26  (WWTP 2) | 48.0 ± 4  (WWTP 1)  88.0 ± 14  (WWTP 2) | 8.5 ± 0.8 *** |  | Germany | Wick, Fink and Ternes, 2010 |
| Carbendazim | - | 154.0 ± 118 **(d)** | 204.0 ± 135 **(d)** | 120.0 ± 79.9 **(d)** | 0.004 **(d)** | 2.0 ± 1.58 **(d)** | Thailand | Juksu *et al.*, 2019 |
| Carbendazim | - |  |  | 14.0 – 103.0 **(b)**  (pre-treatment, primary treatment, conventional activated sludge biological treatment and final decantation) |  |  | Spain | Campos-Mañas *et al.*, 2019 |
| Carbendazim | - | 66.6 ± 1.20 **(a)** | 47.2 **(c)** | 21.6 **(c)** |  |  | China | Liu *et al.*, 2017 |
| Carbendazim | - | 10.0 **(a)**  (River) |  |  |  |  | Romania | Chițescu and Nicolau, 2014 |
| Carbendazim | - | 75.0 **(a)**  (Surface)  50.0 **(a)**  (Groundwater) |  |  |  |  | The Netherlands | Chitescu *et al.*, 2012 |
| Ciclopirox | - |  | 1410.0 | 321.0 |  |  | USA | Bisceglia *et al.*, 2010 |
| Climbazole | - | 1.5 - 158.2 **(b)**  (Rampura canal, Aftabnagar lake, and Dasherkandi Trimohoni) |  |  |  |  | Bangladesh | Angeles *et al.*, 2020 |
| Climbazole | 560 | 13.7 **(d)**  (Xizhijiang River)  82.9 **(d)**  (Shima River) |  |  |  | 26.3 **(d)**  (Xizhijiang River)  37.2 **(d)**  (Shima River) | China | Chen *et al.*, 2014 |
| Climbazole | 560 | 1.0 - 17.2 **(b)** |  |  |  | 0.4 - 96.0 **(b)** | China | Zhang *et al.*, 2015 |
| Climbazole | - | ND  (River)  47.0 ± 4  (Stream 1)  530.0 ± 70  (Stream 2) | 475.0 ± 44  (WWTP 1)  1350.0 ± 70  (WWTP 2) | 312.0 ± 12  (WWTP 1)  443.0 ± 11  (WWTP 2) | 1160.0 ± 80 *** |  | Germany | Wick, Fink and Ternes, 2010 |
| Climbazole | - |  | 23.6 – 187.0 **(b)** * | 11.0 - 97.1 **(b)** * | 0.94 - 12.7 **(b)** * |  | China | Liu *et al.*, 2017 |
| Climbazole | - | ND  (River Reservoir) | 282.0 ± 11 **(d)** | 66.4 ± 0.5 **(d)** | 152.0 ± 5 **(d)**  (Dewatered) | ND | China | Chen *et al.*, 2012 |
| Climbazole | - |  |  |  | 34.0 **(d)**  (Digested Sewage) |  | Spain | Casado *et al.*, 2015 |
| Climbazole | 0.16 |  |  | 150.0 **(c)** |  |  | Europe | Coors *et al.*, 2018 |
| Climbazole | - | 3.0 – 240.0 **(b)** |  | 16.0 – 380.0 **(b)** |  |  | China, India and Philippines | Angeles *et al.*, 2021 |
| Climbazole | - |  |  |  | 165.0 ± 6 **(d)**  (Biosolid in WWTP) |  | China | Chen *et al.*, 2013 |
| Climbazole | - |  |  | 80.0 **(d)**  (Grilling and Sand Speartion, Denifrication, Biological Nitrifcation and Secondary Sedimentation) |  |  | Italy | Montagna *et al.*, 2020 |
| Clotrimazole | - | 0.7 - 29.0 **(b)**  (River) |  | 5.9 - 31.1 **(b)** |  |  | Poland | Zgoła‐Grześkowiak and Grześkowiak, 2013 |
| Clotrimazole | - |  | 256.0 – 1834.0 **(b)** | < MQL – 35.0 **(b)** | 190.0 ± 28 - 2547.0 **(b)**  (Dewatered) | NA – 73.0 **(b)**  (Untreated Solid) | China | Peng *et al.*, 2012 |
| Clotrimazole | - | 1.0 / 1.5 **(e)**  (Groundwater) |  |  |  |  | China | Peng *et al.*, 2014 |
| Clotrimazole | - | <1.0 – 618.0 **(b)**  **(**Rivers, Surface**)**  <1.0 – 191.0 **(b)**  (Groundwater)  <1.0  (Sachet water) |  |  |  |  | Nigeria | Ebele *et al.*, 2020 |
| Clotrimazole | 14.0  (Hospital effluent) |  |  |  |  |  | Scotland | Helwig *et al.*, 2015 |
| Clotrimazole | ≤ 100.0  (Hospital effluent) |  |  |  |  |  | - | Frédéric and Yves, 2014 |
| Clotrimazole | 14.0  (Hospital) |  |  |  |  |  | Switzerland | Escher *et al.*, 2011 |
| Clotrimazole | - |  | 12.0 – 78.0 **(b)** | <LOQ – 6.0 **(b)** |  |  | Switzerland | Kahle *et al.*, 2008 |
| Clotrimazole | - | 4.0  (River) | 33.0 | 8.0 ± 2 **(d)** | 190.0 ± 28 **(d)**  1442.0 ± 107 **(d)** | < MLQ (Estuary)  22.0 (River) | China | Huang *et al.*, 2010 |
| Clotrimazole | 30.0  (River) |  |  |  |  |  | Korea | Lee *et al.*, 2024 |
| Clotrimazole | - | < LOQ | < LOQ | < LOQ | 30.0 – 310.0 **(b)**  (Dewatered Sudge) |  | Sweden | Lindberg, Fick and Tysklind, 2010 |
| Clotrimazole | - |  |  |  | 492.0 ± 21 **(d)**  (Biosolid in WWTP) |  | China | Chen *et al.*, 2013 |
| Clotrimazole | - |  |  |  | 168.4 ± 3.4 - 987.4 ± 224.5 **(b)** |  | Spain | García‐Valcárcel and Tadeo, 2011 |
| Clotrimazole | - |  |  |  | 417.0 **(d)**  (Digested Sewage) |  | Spain | Casado *et al.*, 2015 |
| Clotrimazole | - |  | 8.0 – 67.0 **(b)**  (aqueous + solid) | < LOQ – 1.0 **(b)** | 73.0 - 590.0 **(b)**  (Digested sludge) |  | Sweden | Östman *et al.*, 2017 |
| Clotrimazole | - | 10.7 ± 9.18 **(d)** | 41.0 ± 26.0 **(d)** | 6.4 ± 2.8 **(d)** | 0.7 **(d)** | 37.8 ± 55.3 **(d)** | Thailand | Juksu *et al.*, 2019 |
| Clotrimazole | 1.0 | 0.7 – 103.0 **(b)** |  |  |  | 0.4 - 35.4 **(b)** | China | Zhang *et al.*, 2015 |
| Clotrimazole | - | 91.9 |  |  |  |  | Gambia | Cangola, Abagale and Cobbina, 2024 |
| Clotrimazole | - | 6.0 – 34.0 **(b)**  (River) |  |  |  |  | United Kingdom | Peschka, Roberts and Knepper, 2007 |
| Clotrimazole | - | 250.0 **(d)**  (River)  510.0 **(d)**  (Aquifers) |  |  |  |  | India | Velpandian *et al.*, 2018 |
| Clotrimazole | - | 4.0 ± 1 – 7.0 ± 4 **(b)**  (Stream) |  |  |  |  | Czechia | Grabicova *et al.*, 2015 |
| Clotrimazole | - | 7.0  47.0  (River) | 168.0  27.0 | 18.0  23.0 |  |  | Czechia and Switzerland | Macikova *et al.*, 2014 |
| Clotrimazole | - | 1.0 – 95.0 **(b)**  (Subsurface Coastal Waters) |  |  |  |  | Fiji | Dehm *et al.*, 2021 |
| Clotrimazole | - | 10.0 **(a)**  (River) |  |  |  |  | Romania | Chițescu and Nicolau, 2014 |
| Clotrimazole | - |  |  | 30.0 **(d)** |  |  | South Africa | Assress *et al.*, 2019 |
| Clotrimazole | - |  | 0.3 – 548.0 **(b)** | 0.2 - 53.0 **(b)** |  |  | China | Yang *et al.*, 2022 |
| Clotrimazole | - |  |  | 5.3 **(a)** |  |  | Europe | Loos *et al.*, 2013 |
| Clotrimazole | - |  | <LOQ - 27.0 ± 9 **(b)** | <LOQ – 4.0 ± 1 **(b)** |  |  | Poland | Kotowska and Bieńczyk, 2013 |
| Clotrimazole | - |  | ND - 16.2 **(b)** | ND - 143.3 **(b)** |  |  | South Africa | Assress *et al.*, 2020 |
| Clotrimazole | - |  | 10.0 – 90-0 **(b)** |  |  |  | Slovakia | Fáberová *et al.*, 2017 |
| Clotrimazole | - |  |  | 1.8  3.0  3.9  (Different WWTPs) |  |  | Canada | Westlund and Yargeau, 2017 |
| Clotrimazole | - |  | LOD – 650.0 **(b)**  LOQ – 500.0 **(b)**  LOQ – 900.0 **(b)**  (Different WWTPs) | LOD – 8650.0 **(b)**  LOQ – 300.0 **(b)**  LOQ – 1200.0 **(b)**  (Different WWTPs) |  |  | Ireland | Lacey *et al.*, 2012 |
| Clotrimazole | - | ND  (River Reservoir) | 6.7 ± 0.1 **(d)** | 2.7 ± 0.1 **(d)** | 426.0 ± 16 **(d)**  (Dewatered) | ND | China | Chen *et al.*, 2012 |
| Clotrimazole | - | 23.0 **(a)**  (River) | 231.0 **(a)** |  |  |  | Egypt | Abou-Elwafa Abdallah *et al.*, 2019 |
| Clotrimazole | - | 9.0 ± 2 **(a)**  (River) | 80.0 ± 3 **(a)** | 15.0 ± 2 **(a)** |  |  | Spain | Casado *et al.*, 2014 |
| Clotrimazole | - | ND - 5.7 **(b)**  (River) |  |  |  | 44.7 ± 17.5 **(a)** | China | Huang *et al.*, 2013 |
| Clotrimazole | - |  |  |  |  | 9.22 **(d)**  (Xizhijiang River)  18.2 **(d)**  (Shima River) | China | Chen *et al.*, 2014 |
| Clotrimazole | - | ND | ND | ND |  |  | England | Wattanayon and Kasprzyk-Hordern, 2021 |
| Clotrimazole | - | 83.0 **(a)** |  | 220.0 **(a)** | <LOQ |  | Sweden | Fick and Lindberg, 2014 |
| Clotrimazole | - |  | ND – 98.5 **(b)** | ND |  |  | China | Shi *et al.*, 2020 |
| Cyproconazole | - | 13.8 / 69.1 **(e)**  (River) | 313.6 / 1735 **(e)** | 169.3 / 836.4 **(e)**  (Secondary Treatment)  112.5 / 349.4 **(e)**  (Tertiary Treatment) |  |  | Greece | Stamatis, Hela and Konstantinou, 2010 |
| Difenoconazole | - | < LOQ  (Reservoir) |  |  |  |  | Brazil | López-Doval *et al.*, 2017 |
| Dimentomorph | - | 6.2 - 16.3 **(b)**  (Watershed) |  |  |  |  | Cameroon | Branchet *et al.*, 2018 |
| Econazole | - | < LOQ | < LOQ | < LOQ | 210.0 – 1000.0 **(b)**  (Digested Dewatered Sludge) |  | Sweden | Lindberg, Fick and Tysklind, 2010 |
| Econazole | - |  | 1.2 **(a)** | 0.51 **(a)** | 120.8  (Dewatered) | 8.3  (Untreated solid) | China | Huang *et al.*, 2012 |
| Econazole | - | ND – 0.9 **(b)**  (River) |  |  |  | 55.8 ± 5.7 **(a)** | China | Huang *et al.*, 2013 |
| Econazole | - | < MLQ  (River) | < LOQ | < LOQ | 54.0 ± 8 **(d)**  140.0 ± 4 **(d)**  (sewage) | 1.0 (Estuary)  4.0 (River) | China | Huang *et al.*, 2010 |
| Econazole | - |  |  |  | 2.1 ± 0.4 - 27.2 ± 0.4 **(b)** |  | Spain | García‐Valcárcel and Tadeo, 2011 |
| Econazole | - |  | <LOQ – 89.0 **(b)**  (aqueous + solid) | < LOQ | 73.0 – 660.0 **(b)**  (Digested sludge) |  | Sweden | Östman *et al.*, 2017 |
| Econazole | - |  | 5.5 **(a)** | < LOQ |  |  | Sweden | Östman *et al.*, 2019 |
| Econazole | - |  | 0.06 - 118.8 **(b)** | <LOD – 45.9 **(b)** |  |  | China | Yang *et al.*, 2022 |
| Econazole | - | 16.4 ± 0.6 **(a)** | 18.1 ± 1.2 **(a)** | 5.6 ± 0.7 **(a)** |  |  | China | Wang *et al.*, 2018 |
| Econazole | - |  | ND - 4.8 **(b)** | ND - 20.1 **(b)** |  |  | South Africa | Assress *et al.*, 2020 |
| Econazole | - |  | 6.0 – 82.0 **(b)** | < 1.1 | 54.0 ± 8 – 153.0 **(b)**  (Dewatered) | 5.0 – 17.0 **(b)**  (Untreated Solid) | China | Peng *et al.*, 2012 |
| Econazole | - | ND | ND | ND |  |  | England | Wattanayon and Kasprzyk-Hordern, 2021 |
| Epocixonazole | - | 2.3 - 4.5 **(b)**  (Watershed) |  |  |  |  | Cameroon | Branchet *et al.*, 2018 |
| Epoxiconazole | - | 16.0 ± 1.1 | 48.0 ± 3.8 | 18.2 ± 0.6 |  |  | China | Zhao *et al.*, 2018 |
| Epoxiconazole |  | 0.8 **(a)**  (Reservoir) |  |  |  |  | Brazil | López-Doval *et al.*, 2017 |
| Epoxiconazole | - |  |  |  | < LOQ - 20.9 ± 4.3 **(b)** |  | Spain | García‐Valcárcel and Tadeo, 2011 |
| Epoxiconazole | - | 67.3 ± 26.5 **(d)** | ND | ND |  |  | England | Wattanayon and Kasprzyk-Hordern, 2021 |
| Epoxiconazole | - | 2700.0 **(a)**  (River) |  |  |  |  | Germany | Berenzen *et al.*, 2005 |
| Ethylparaben | - | <0.3 – 1.6 **(b)**  (River) | 2.2 – 719.0 **(b)** | <0.3 – 17.0 **(b)**  (Secondary Treatment) |  |  | Switzerland | Jonkers *et al.*, 2009 |
| Ethylparaben | 23000.0 | **ND**  (Xizhijiang River)  43.1 **(d)**  (Shima River) |  |  |  |  | China | Chen *et al.*, 2014 |
| Fenbuconazole | - |  |  |  | 3.3  (Sewage) |  | Greece | Miserli, Nastopoulou and Konstantinou, 2022 |
| Fenticonazole | - |  |  |  | 110.0 **(d)** |  | Spain | Castro *et al.*, 2016 |
| Fluconazole | - |  | 90.0 - 570.0 **(b)** | 100.0 – 140.0 **(b)** | < LOQ  (Digested Dewatered Sludge) |  | Sweden | Lindberg, Fick and Tysklind, 2010 |
| Fluconazole | - |  | 110.0 – 170.0 **(b)** | 110.0 – 170.0 **(b)** |  |  | China | Qi *et al.*, 2015 |
| Fluconazole | - |  | 5.0 – 358.0 **(b)** | 5.0 – 308.0 **(b)** |  |  | Germany | Rossmann *et al.*, 2014 |
| Fluconazole | - | < LOQ - 9.0 **(b)**  (Lake) | 32.0 – 109.0 **(b)** | 28.0 – 83.0 **(b)** |  |  | Switzerland | Kahle *et al.*, 2008 |
| Fluconazole | - | 21.7 / 56.2 **(e)**  (Groundwater) |  |  |  |  | China | Peng *et al.*, 2014 |
| Fluconazole | - | 124.0 **(a)**  (Groundwater) |  |  |  |  | USA | Elliott *et al.*, 2018 |
| Fluconazole | - |  | 167.0 ± 28 **(a)**  (Hospital) | 164.0 ± 43 **(a)**  (Hospital) |  |  | Germany | Knoth *et al.*, 2018 |
| Fluconazole | - | 68.1±1.75 **(a)** | 78.6 **(c)** | 82.7 **(c)** |  |  | China | Yang *et al.*, 2017 |
| Fluconazole | - | ND - 109.6 **(b)**  (River) |  |  |  |  | China | Huang *et al.*, 2013 |
| Fluconazole | - | 19.7 **(d)**  (Xizhijiang River)  52.8 **(d)**  (Shima River) |  |  |  |  | China | Chen *et al.*, 2014 |
| Fluconazole | - | 111.0 ± 13 **(a)**  (River) |  |  |  |  | Korea | Kim *et al.*, 2009 |
| Fluconazole | - | 178.0 - 271.0  (River) |  |  |  |  | South Africa | Monapathi *et al.*, 2021 |
| Fluconazole | - | 32.0 ± 4 **(a)**  (River) | 93.0 ± 17 **(a)** | 95.0 ± 9 **(a)** |  |  | Spain | Casado *et al.*, 2014 |
| Fluconazole | - |  | 22.0 – 170.0 **(b)** | 50.0 – 139.0 **(b)** |  |  | China | Peng *et al.*, 2012 |
| Fluconazole | - |  | 51.6 | 38.1 - 44.3 **(b)** | 4770.0  (Raw Sewage)  960.0 - 2010.0 **(b)**  (Treated Sewage) |  | China | Wroński, Trawiński and Skibiński, 2024 |
| Fluconazole | - |  | 8.4 - 60.5 **(b)** * | 13.1 - 63.6 **(b)** * | 0.01 - 0.1 **(b)** * |  | China | Liu *et al.*, 2017 |
| Fluconazole | - |  | ND | ND |  |  | Malaysia | Hanafiah *et al.*, 2024 |
| Fluconazole | - |  |  |  | < LOQ - 57.5 ± 1.0 **(b)** |  | Spain | García‐Valcárcel and Tadeo, 2011 |
| Fluconazole | - | 14.7 - 898.8 **(b)**  (River, surface) |  |  |  |  | Bangladesh | Angeles *et al.*, 2020 |
| Fluconazole | 613 | 2.8 - 13.6 **(b)** |  |  |  | ND | China | Zhang *et al.*, 2015 |
| Fluconazole | - |  | 236950000.0 **(a)** |  |  |  | India | Lübbert *et al.*, 2017 |
| Fluconazole | - | 1060.0 **(d)**  (River)  13200.0 **(d)**  (Aquifers) |  |  |  |  | India | Velpandian *et al.*, 2018 |
| Fluconazole | - |  |  | 380.0 **(d)**  (Grilling and Sand Speartion, Denifrication, Biological Nitrifcation and Secondary Sedimentation) |  |  | Italy | Montagna *et al.*, 2020 |
| Fluconazole | - | 98.7 **(a)**  (River) |  |  |  |  | Brazil | de Barros *et al.*, 2018 |
| Fluconazole | - | 0.3 - 9.3 **(b)**  (River) |  | 6.0 – 45.0 **(b)** |  |  | Canada | Elkayar *et al.*, 2022 |
| Fluconazole | - | 2.9 **(a)**  (Lake) |  |  |  |  | China | Asghar *et al.*, 2018 |
| Fluconazole | - | 6.7 ± 1.0 **(a)** | 8.2 ± 0.5 **(a)** | ND |  |  | China | Wang *et al.*, 2018 |
| Fluconazole |  | 27.0  18.0  (River) | 6426.0  4640.0 | 200.0  182.0 |  |  | Czechia and Switzerland | Macikova *et al.*, 2014 |
| Fluconazole | - | 0.5 – 22.0 **(b)**  (Subsurface Coastal Waters) |  |  |  |  | Fiji | Dehm *et al.*, 2021 |
| Fluconazole | - | 150.0 **(a)** |  | 16230.0 **(a)** |  |  | Nigeria | Hu *et al.*, 2021 |
| Fluconazole | - | <10.0 **(a)**  (River) |  |  |  |  | Romania | Chițescu and Nicolau, 2014 |
| Fluconazole | - | 50.0 **(a)**  (Surface) |  |  |  |  | The Netherlands | Chitescu *et al.*, 2012 |
| Fluconazole | - |  |  | 302.4 **(d)** |  |  | South Africa | Assress *et al.*, 2019 |
| Fluconazole | - |  | < LOD - 8.3 **(b)** | <LOD - 3.2 **(b)** |  |  | China | Yang *et al.*, 2022 |
| Fluconazole | - | <MQL  (River) | ND | 101.0 ± 35.6 **(d)** |  |  | England | Wattanayon and Kasprzyk-Hordern, 2021 |
| Fluconazole | - |  |  | 598 **(a)** |  |  | Europe | Loos *et al.*, 2013 |
| Fluconazole | 9.46 |  |  | 44.0 **(c)** |  |  | Europe | Coors *et al.*, 2018 |
| Fluconazole | - |  | 158.5 - 167.5 **(b)** | 151.3 - 163.0 **(b)** |  |  | Germany | Gurke *et al.*, 2015 |
| Fluconazole | - |  | 119.2 – 9959.0 **(b)** | 89.2 - 3341.8 **(b)**  (WWTPs)  407.9  (Hospital) |  |  | South Africa | Assress *et al.*, 2020 |
| Fluconazole | - |  |  | 109480.0 **(a)** |  |  | Spain | Lopez-Herguedas *et al.*, 2022 |
| Fluconazole | - |  |  | 122.0 – 372.0 **(b)** |  |  | Sweden | Kårelid, Larsson and Björlenius, 2017b |
| Fluconazole | - |  |  | 95.0 – 379.0 **(b)** |  |  | Sweden | Kårelid, Larsson and Björlenius, 2017a |
| Fluconazole | - | 75.7 ± 68.5 **(d)** | 102.0 ± 73.6 **(d)** | 101.0 ± 73.7 **(d)** | 0,0029 **(d)** | 0.6 ± 0.33 **(d)** | Thailand | Juksu *et al.*, 2019 |
| Fluconazole | - | < MQL – 1413.0 **(b)**  (River)  < MQL –1294.0 **(b)**  (Reservoir) |  |  |  |  | Brazil | Reis *et al.*, 2019 |
| Fluconazole | - | ND  (River Reservoir) | 65.1 ± 1.5 **(d)** | 61.1 ± 1.4 **(d)** | 8.2 ± 0.6 **(d)**  (Dewatered) | ND | China | Chen *et al.*, 2012 |
| Fluconazole | - |  | 20-0 – 1710.0 **(b)** |  |  |  | Slovakia | Fáberová *et al.*, 2017 |
| Fluconazole | - |  | 21.4 ± 7.4 |  |  |  | Spain | Sabater-Liesa *et al.*, 2021 |
| Fluconazole | - |  |  | 1348.0  27606.0  23324.0  (Different WWTPs) |  |  | Canada | Westlund and Yargeau, 2017 |
| Fluconazole | - | <LOQ |  | <LOQ | 160 **(a)** |  | Sweden | Fick and Lindberg, 2014 |
| Fluconazole | - |  | 30.0 - 333.0 **(b)**  (aqueous + solid) | < LOQ - 170.0 **(b)** | < LOQ  (Digested sludge) |  | Sweden | Östman *et al.*, 2017 |
| Fluconazole | - |  | 380.0 **(a)** | 430.0 **(a)** |  |  | Sweden | Östman *et al.*, 2019 |
| Fluconazole | - |  | 17.7 | 15.5  (Terciary Treatment) |  |  | Sweden | Burzio *et al.*, 2022 |
| Fluconazole | - |  | ND – 61.8 **(b)** | ND – 4.4 **(b)** |  |  | China | Shi *et al.*, 2020 |
| Fluconazole |  | 330.7  (Surface)  174.6  (Groundwater) |  |  |  |  | USA | Bradley *et al.*, 2016 |
| Griseofluvin | - | 7.3 / 15.7 **(e)**  (River) |  |  |  |  | China | Wang *et al.*, 2015 |
| Griseofluvin | - | 5.0 **(a)**  (River) |  |  |  |  | Romania | Chițescu and Nicolau, 2014 |
| Griseofluvin | - | <LOQ - 2.9 **(b)**  (River) |  |  |  |  | Vietnam | Ngo *et al.*, 2021 |
| Griseofluvin | - | 2.0 – 230.0 **(b)** |  | 25.0 - 140.0 **(b)** |  |  | India and USA | Angeles *et al.*, 2021 |
| Griseofluvin | - |  | 38.0 |  |  |  | Vietnam | Kuroda *et al.*, 2015 |
| Hexaconazole | - | 1.2  (Rampura Canal, Aftabnagar Lake, and Dasherkandi Trimohoni) |  |  |  |  | Bangladesh | Angeles *et al.*, 2020 |
| Hexaconazole | - | 8.3 ± 0.9 | 11.9 ± 0.3 | 11.7 ± 2.3 |  |  | China | Zhao *et al.*, 2018 |
| Hexaconazole | - |  |  |  | < LOQ - 4.8 ± 0.37 **(b)** |  | Spain | García‐Valcárcel and Tadeo, 2011 |
| Imazalil | - |  |  | 7038.0 - 19802.0 **(b)**  sequencing batch reactor (SBR) with activated sludge (industrial wastewater)  3.0 – 496.0 **(b)**  pre-treatment, primary treatment, conventional activated sludge biological treatment and final decantation  (urban wastewater) |  |  | Spain | Campos-Mañas *et al.*, 2019 |
| Imazalil | - | 4900.0 **(a)**  (River) |  |  |  |  | Spain | Bijlsma *et al.*, 2021 |
| Imazalil | - | 22.0 |  | 8.0 - 150.0 **(b)** |  |  | China and Sweden | Angeles *et al.*, 2021 |
| Imazalil | - | 49.1 - 409.8 **(b)**  (River) |  |  |  |  | Spain | Ccanccapa *et al.*, 2016 |
| Imazalil | - |  |  |  | 31.0 **(d)** |  | Spain | Castro *et al.*, 2016 |
| Imazalil | - | 185 **(a)** |  |  |  |  | Germany | Wluka *et al.*, 2016 |
| Itraconazole | - |  | 6000.0 | 15.0 |  |  | United Kingdom | Van De Steene and Lambert, 2008 |
| Itraconazole | - |  |  |  | 204.0 **(d)** |  | Spain | Castro *et al.*, 2016 |
| Itraconazole | - | ND | ND | ND | 0.4 **(d)** | 25.4 ± 41.2 **(d)** | Thailand | Juksu *et al.*, 2019 |
| Itraconazole | - | 20.0 **(d)**  (River)  10.0 **(d)**  (Aquifer) |  |  |  |  | India | Velpandian *et al.*, 2018 |
| Itraconazole | - | < LOD | 28.4 **(a)** | < LOQ |  |  | Belgium | Van De Steene, Stove and Lambert, 2010 |
| Itraconazole | - | 3.9 ± 0.2 **(a)** | 6.2 ± 0.5 **(a)** | ND |  |  | China | Wang *et al.*, 2018 |
| Itraconazole | - |  |  | 2.4 **(d)** |  |  | South Africa | Assress *et al.*, 2019 |
| Itraconazole | - |  | ND | 23.7 – 4666.0 **(b)**  (WWTPs)  8.2  (Hospital) |  |  | South Africa | Assress *et al.*, 2020 |
| Itraconazole | - |  | 0.2 - 5.8 **(b)** * | 0.07 - 0.4 **(b)** * | 0.1 - 8.1 **(b)** * |  | China | Liu *et al.*, 2017 |
| Ketoconazole | - |  | 30.0 | < LOQ | 280.0 – 1800.0  (Dewatered Sludge) |  | Sweden | Lindberg, Fick and Tysklind, 2010 |
| Ketoconazole | - |  | 41.0 – 384.0  (raw wastewater)  66.0 – 856.0  (influent) | ND – 85.0 | 126.0 - 490.0 **(b)**  (Dewatered) | 35.0 - 268.0 **(b)**  (Untreated Solid) | China | Peng *et al.*, 2012 |
| Ketoconazole | 50  (Hospital effluent) |  |  | Hospital |  |  | Scotland | Helwig *et al.*, 2015 |
| Ketoconazole | - |  | 348.0 ±179 **(a)**  (Hospital) | 211.0 ± 294 **(a)**  (Hospital) |  |  | Sweden | Knoth *et al.*, 2018 |
| Ketoconazole | - | < LOD | 66.6 **(a)** | 34.8 **(a)** |  |  | Belgium | Van De Steene, Stove and Lambert, 2010 |
| Ketoconazole | - | 25.8 ± 0.28 **(a)** | 15.2 **(c)** | 2.3 **(c)** |  |  | China | Yang *et al.*, 2017 |
| Ketoconazole | - |  | 143000.0 | 1120.0 |  |  | United Kingdom | Van De Steene and Lambert, 2008 |
| Ketoconazole | - |  |  |  | 20.0 - 2507.0 **(b)** |  | Spain | Castro *et al.*, 2021 |
| Ketoconazole | - | 1.0  (River) | 16.0 | 2.0 **(d)** | 437.0 ± 137 **(d)**  194.0 ± 3 **(d)**  (Sewage) | 2.0 (Estuary)  5.0 (River) | China | Huang *et al.*, 2010 |
| Ketoconazole | - |  |  |  | 317.0 ± 0.06 **(a)**  (sewage) |  | Brazil | Cerqueira *et al.*, 2018 |
| Ketoconazole | - |  |  |  | 61.9 ± 3.6 - 4448.9 ± 81.4 **(b)** |  | Spain | García‐Valcárcel and Tadeo, 2011 |
| Ketoconazole | - | 23.0 ± 0.5 **(a)**  (River) | 27.1 ± 0.3 **(a)** | ND |  |  | China | Wang *et al.*, 2018 |
| Ketoconazole | - | <1.0  <1.0  (River) | 77.0  142.0 | 6.0  15.0 |  |  | Czechia and Switzerland | Macikova *et al.*, 2014 |
| Ketoconazole | - | 10.0 – 620.0 **(b)**  (Surface – Near STP) |  |  |  |  | Fiji | Dehm *et al.*, 2021 |
| Ketoconazole | - | 4.0 – 11.0 **(b)**  (River) | 55.0 – 191.0 **(b)** | 10.0 – 36.0 **(b)**;  LOQ **(a)**  (Primary and Biological Treatment) |  |  | Spain | Casado *et al.*, 2014 |
| Ketoconazole | - | 75.0 **(a)**  (Surface)  50.0 **(a)**  (Groundwater) |  |  |  |  | The Netherlands | Chitescu *et al.*, 2012 |
| Ketoconazole | - | ND – 1.5 **(b)**  (River) |  |  |  | 9.3 ± 0.7 **(a)** | China | Huang *et al.*, 2013 |
| Ketoconazole | - |  | ND – 66.6 **(b)** | 6.7 – 9.0 **(b)** |  |  | South Africa | Assress *et al.*, 2020 |
| Ketoconazole | - | ND | ND | ND |  |  | England | Wattanayon and Kasprzyk-Hordern, 2021 |
| Ketoconazole | - |  | < LOQ - 526.0 **(b)**  (aqueous + solid) | < LOQ - 41.0 **(b)** | < LOQ - 10000.0 **(b)**  (Digested sludge) |  | Sweden | Östman *et al.*, 2017 |
| Ketoconazole | - |  | 200.2 | 12.4  (Terciary Treatment) |  |  | Sweden | Burzio *et al.*, 2022 |
| Ketoconazole | - |  | 109.0 **(a)** |  |  |  | United Arab Emirates | Maraqa, Meetani and Alhalabi, 2020 |
| Ketoconazole | - | 124.7 **(a)** | 223.7 **(a)** | 572.6 **(a)** |  |  | South Africa | Holton *et al.*, 2022 |
| Ketoconazole | - |  | 91.6 **(a)** | 12.4 **(a)** | 231.9  (Dewatered) | 230.9  (Untreated solid) | China | Huang *et al.*, 2012 |
| Ketoconazole | - | 140.0 **(d)**  (River)  190.0 **(d)**  (Aquifers) |  |  |  |  | India | Velpandian *et al.*, 2018 |
| Ketoconazole | - |  |  | 12.3 **(d)** |  |  | South Africa | Assress *et al.*, 2019 |
| Ketoconazole | - |  |  |  | 185.0 **(d)**  (Digested Sewage) |  | Spain | Casado *et al.*, 2015 |
| Ketoconazole | - |  | 1.2 - 28.2 **(b)** * | 0.04 - 1.6 **(b)** * | 0.2 - 7.0 **(b)** * |  | China | Liu *et al.*, 2017 |
| Ketoconazole | - | ND  (River)  < LOQ  (Stream 1)  < LOQ  (Stream 2) | < LOQ  (WWTP 1)  90.0 ± 15  (WWTP 2) | < LOQ  (WWTP 1)  < LOQ  (WWTP 2) | 328.0 ± 45 *** |  | Germany | Wick, Fink and Ternes, 2010 |
| Ketoconazole | - | 8.03 ± 4.47 **(d)** | 52.9 ± 54.7 **(d)** | 4.3 ± 3.38 **(d)** | 0.6 **(d)** | 58.7 ± 86.6 **(d)** | Thailand | Juksu *et al.*, 2019 |
| Ketoconazole | - |  |  |  |  | ND  (Xizhijiang River)  ND  (Shima River) | China | Chen *et al.*, 2014 |
| Ketoconazole | - |  |  | < LOQ |  |  | Spain | Lopez-Herguedas *et al.*, 2022 |
| Ketoconazole | - | 8.5  (Surface)  ND  (Groundwater) |  |  |  |  | USA | Bradley *et al.*, 2016 |
| Metalaxyl | - | 7.6 - 16.7 **(b)**  (River) |  |  |  |  | Cameroon | Branchet *et al.*, 2018 |
| Metalaxyl | - | 1.1 - 30.8 **(b)**  (River) |  |  |  |  | China | Peng *et al.*, 2018 |
| Methfuroxam | - | 2.0 – 17.0 **(b)** |  | 4.0 – 6.0 **(b)** |  |  | China | Angeles *et al.*, 2021 |
| Methylparaben | - | 96.0 **(a)**  (Lagoon) |  |  |  |  | Nigeria | Folarin *et al.*, 2020 |
| Methylparaben | - | 3.1 – 17.0 **(b)**  (River) | 65.0 – 9880.0 **(b)** | 4.6 – 423.0 **(b)**  (Secondary Treatment) |  |  | Switzerland | Jonkers *et al.*, 2009 |
| Methylparaben | - |  | 1014.0 – 33580.0 **(b)** |  |  |  | India | Ravichandran, Yoganathan and Philip, 2021 |
| Methylparaben | 15000.0 | 7.7 **(d)**  (Xizhijiang River)  21.8 **(d)**  (Shima River) |  |  |  | 14.8 **(d)**  (Xizhijiang River)  16.0 **(d)**  (Shima River) | China | Chen *et al.*, 2014 |
| Metronidazole | - | 4.9 - 35.6 **(b)**  (Groundwater – Near WW Discharge) |  |  |  |  | Taiwan | Lin *et al.*, 2015 |
| Metronidazole | - |  | 25.0 – 56.0 **(b)**  (WWTP) | 330.0 – 1640.0 **(b)**  (hospital)  13.0 – 41.0 **(b)**  (WWTP) |  |  | Italy | Verlicchi *et al.*, 2012 |
| Metronidazole | 13 |  | Hospital |  |  |  | Turkey | Yilmaz *et al.*, 2017 |
| Metronidazole | - | 0.05 - 13.5 (b)  (River) |  |  |  |  | India | Hossain *et al.*, 2018 |
| Metronidazole | - | 20.0 **(d)**  (River)  90.0 **(d)**  (Aquifer) |  |  |  |  | India | Velpandian *et al.*, 2018 |
| Metronidazole | - |  | 113.0 **(a)** | 12315.0 **(a)**  (hospital)  19.4 – 83.5 **(b)**  (WWTP) |  |  | Portugal | Santos *et al.*, 2013 |
| Metronidazole | 125 |  | 164264.4 **(a)** |  | 18.0 |  | South Africa | Faleye *et al.*, 2019 |
| Metronidazole |  | 148.7 **(a)** | 1936.5 **(a)** | 100.6 **(a)** |  |  | South Africa | Holton *et al.*, 2022 |
| Metronidazole | - | 1.1 **(a)**  (River) |  |  |  |  | China | Asghar *et al.*, 2018 |
| Metronidazole | - |  | 51.0 **(a)** | 39.0 **(a)** |  |  | Sweden | Östman *et al.*, 2019 |
| Miconazole | - |  | <LOQ | <LOQ | 160.0 – 970.0 **(b)**  (Digested Dewatered Sludge) |  | Sweden | Lindberg, Fick and Tysklind, 2010 |
| Miconazole | - |  | 241.0 – 1086.0 **(b)**  (raw wastewater)  76.0 – 227.0 **(b)**  (influent) | 6.0 – 26.0 **(b)** | 240.0 ± 41 - 2069.0 **(b)**  (Dewatered) | 40.0 - 190.0 **(b)**  (Untreated Solid) | China | Peng *et al.*, 2012 |
| Miconazole | - | 2.8 / 6.7 **(e)**  (Groundwater) |  |  |  |  | China | Peng *et al.*, 2014 |
| Miconazole | - | 27.3 ± 0.56 **(a)** | 3.8 **(c)** | 26.0 **(c)** |  |  | China | Yang *et al.*, 2017 |
| Miconazole | - |  | 15000.0 | 81.0 |  |  | United Kingdom | Van De Steene and Lambert, 2008 |
| Miconazole | - |  |  |  | 88.0 – 4922.0 **(b)** |  | Spain | Castro *et al.*, 2021 |
| Miconazole | - | 2.0  (River) | 32.0 | 3.0 ± 1 **(d)** | 238.0 ± 41 **(d)**  1405.0 ± 96 **(d)**  (Sewage) | 1.0 (Estuary)  35.0 (River) | China | Huang *et al.*, 2010 |
| Miconazole | - |  | 3.1 – 459.0 **(b)** * | 0.2 - 5.1 **(b)** * | 4.0 – 133.0 **(b)** * |  | China | Liu *et al.*, 2017 |
| Miconazole | - |  | ND | ND |  |  | Malaysia | Hanafiah *et al.*, 2024 |
| Miconazole | - |  | 5.2 - 43.0 **(b)** | 1.6 - 25.0 **(b)** |  |  | Canada | Guerra *et al.*, 2014 |
| Miconazole | - |  |  |  | 515 ± 0.04 **(a)**  (sewage) |  | Brazil | Cerqueira *et al.*, 2018 |
| Miconazole | - |  |  |  | 39.3 - 317.0 **(b)**  (domestic WWTP) |  | South Korea | Subedi *et al.*, 2014 |
| Miconazole | - | 116.9 **(a)**  (River) |  |  |  |  | Brazil | de Barros *et al.*, 2018 |
| Miconazole | - | ND |  |  | 5.0 **(d)**  (Xizhijiang River)  8.6 **(d)**  (Shima River) |  | China | Chen *et al.*, 2014 |
| Miconazole | - | < 1.0  < 1.0  (River) | < 1.0  15.0 | < 1.0  < 1.0 |  |  | Czechia and Switzerland | Macikova *et al.*, 2014 |
| Miconazole | - |  |  |  |  | 10.0  (Surface - Coast) | USA | Maruya *et al.*, 2015 |
| Miconazole | - | 1450.0 **(d)**  (River)  78.0 **(d)**  (Aquifer) |  |  |  |  | India | Velpandian *et al.*, 2018 |
| Miconazole | - |  | 67.0 **(d)**  (STP1)  42.0 **(d)**  (STP2) | 8.0 **(d)**  (STP1)  25.0 **(d)**  (STP2) | 240.0 **(d)**  (STP1)  240.0 **(d)**  (STP2) |  | India | Subedi *et al.*, 2017 |
| Miconazole | - | 0.6 - 38.9 **(b)**  (River) |  |  |  | 69.7 ± 15.5 **(a)** | China | Huang *et al.*, 2013 |
| Miconazole | - | ND |  | 48.0 |  |  | China | Angeles *et al.*, 2021 |
| Miconazole | - | < LOD | 337.9 **(a)** | 35.7 **(a)** |  |  | Belgium | Van De Steene, Stove and Lambert, 2010 |
| Miconazole | - |  |  | 14.8 **(a)** |  |  | Europe | Loos *et al.*, 2013 |
| Miconazole | - |  | < LOQ - 60.0 **(b)**  (aqueous + solid) | < LOQ | < LOQ – 700.0 **(b)**  (Digested sludge) |  | Sweden | Östman *et al.*, 2017 |
| Miconazole | - |  |  |  | 427.0 ± 25 **(d)**  (Biosolid in WWTP) |  | China | Chen *et al.*, 2013 |
| Miconazole | - |  | 11.3 **(a)** | 0.87 **(a)** | 1258.0 (Dewatered) | 87.9  (Untreated solid) | China | Huang *et al.*, 2012 |
| Miconazole | - | 18.5 ± 1.1 **(a)** | 22.1 ± 0.8 **(a)** | 3.4 ± 0.3 **(a)** |  |  | China | Wang *et al.*, 2018 |
| Miconazole | - |  | ND - 447.0 **(b)** | ND - 58.7 (**b)** |  |  | China | Shi *et al.*, 2020 |
| Miconazole | - | ND  (River Reservoir) | 1.1 ± 0.1 **(d)** | 0.5 ± 0.1 **(d)** | 150.0 ± 19 **(d)**  (Dewatered) | ND | China | Chen *et al.*, 2012 |
| Miconazole | - |  | 1410.0 | 1020.0 | 327.0 |  | India | Subedi *et al.*, 2015 |
| Miconazole | - |  | 80.0 ± 20 **(a)** | 15.0 ± 2 **(a)**  (Primary and Biological Treatment) |  |  | Spain | Casado *et al.*, 2014 |
| Miconazole | - |  |  |  | 141.0 **(d)**  (Digested Sewage) |  | Spain | Casado *et al.*, 2015 |
| Miconazole | - |  | ND - 16.7 **(b)** | ND - 16.4 **(b)** |  |  | South Africa | Assress *et al.*, 2020 |
| Miconazole | - | 6.2 ± 4.99 **(d)** | 20.0 ± 16.2 **(d)** | 3.2 ± 1.99 **(d)** | 0.5 **(d)** | 34.0 ± 54.3 **(d)** | Thailand | (Juksu *et al.*, 2019) |
| Miconazole | - | ND | ND | ND |  |  | England | Wattanayon and Kasprzyk-Hordern, 2021 |
| Naftifine | - |  | <LOD – 24.6 **(b)** | <LOD – 18.4 **(b)** |  |  | China | Yang *et al.*, 2022 |
| Parabens | - |  | 600.0 – 3700.0 **(b)**  (WWTP 1)  660.0 – 3900.0**(b)**  (WWTP 2) | 8.5 – 100.0 **(b)**  (WWTP 1)  (Anoxic/Oxic (A/O) Processes)  8.7 – 70.0 **(b)**  (WWTP 2)  (Biological Selectors and UV Treatment) |  |  | China | Li *et al.*, 2018 |
| Penconazole | - | 3.9 - 46.9 **(b)**  (River) |  |  |  |  | Cameroon | Branchet *et al.*, 2018 |
| Penconazole | - | 8.8 / 27.2 **(e)**  (River) | 47.2 / 155.2 **(e)** | 22.2 / 79.5 **(e)** (Secondary Treatment)  13.0 / 45.1 **(e)**  (Tertiary Treatment) |  |  | Greece | Stamatis, Hela and Konstantinou, 2010 |
| Prochloraze | - | 0.0  (River) |  |  |  |  | Cameroon | Branchet *et al.*, 2018 |
| Propiconazole | - |  | < LOQ - 14.0 **(b)**  (aqueous + solid) | < LOQ - 10.0 **(b)** | < LOQ - 120.0 **(b)**  (Digested sludge) |  | Sweden | Östman *et al.*, 2017 |
| Propiconazole | - |  |  |  | <LOQ – 32.1 ± 1.6 **(b)** |  | Spain | García‐Valcárcel and Tadeo, 2011 |
| Propiconazole | - | 0.3 / 0.8 **(e)**  (Groundwater) |  |  |  |  | China | Peng *et al.*, 2014 |
| Propiconazole | - |  | 160.0 | 82.0 |  |  | United Kingdom | Van De Steene and Lambert, 2008 |
| Propiconazole | - | 1. 2 - 5.2 **(b)**  (River, Lake and Canal) |  |  |  |  | Bangladesh | Angeles *et al.*, 2020 |
| Propiconazole | - | 1.8 - 810.4 **(b)**  (River) |  |  |  |  | China | Peng *et al.*, 2018 |
| Propiconazole | - | 6.6  (River) | < MLQ | 2.5 ± 0.4 **(d)** | < MLQ  (Sewage) | ND | China | Huang *et al.*, 2010 |
| Propiconazole | 10 | 178.3 **(a)**  (Surface – Near WWTP Discharge) | 3889.3 **(a)** | 3561.6 **(a)** |  |  | Belgium | Van De Steene, Stove and Lambert, 2010 |
| Propiconazole | - |  | 4.0 – 27.0 **(b)** | 5.0 – 40.0 **(b)** |  |  | Switzerland | Kahle *et al.*, 2008 |
| Propiconazole | - |  |  | 1815.0  7.3  14.7  (Different WWTPs) |  |  | Canada | Westlund and Yargeau, 2017 |
| Propiconazole | - | 5.1 ± 0.5  (River)  5.6 ± 1.4  (Stream 1)  6.0 ± 0.6  (Stream 2) | 16.0 ± 4  (WWTP 1)  < LOQ  (WWTP 2) | 14.0 ± 1  (WWTP 1)  10.0 ± 2  (WWTP 2) | 12.0 ± 2 *** |  | Germany | Wick, Fink and Ternes, 2010 |
| Propiconazole | 6.8 |  |  | 12.0 **(c)** |  |  | Europe | Coors *et al.*, 2018 |
| Propiconazole | - | ND – 32.6 **(b)**  (River) |  |  |  | 0.8 ± 0.1 **(a)** | China | Huang *et al.*, 2013 |
| Propiconazole | - | <0.25 – 21.8 **(b)**  (River) |  |  |  |  | Spain | Rico *et al.*, 2019 |
| Propiconazole | - |  |  | 5.0 – 47.0 **(b)** |  |  | Canada | Elkayar *et al.*, 2022 |
| Propiconazole | - | 41.3 ± 0.9 **(d)** | ND | ND |  |  | England | Wattanayon and Kasprzyk-Hordern, 2021 |
| Propiconazole | - | 25.0 (a)  (Surface) |  |  |  |  | The Netherlands | Chitescu *et al.*, 2012 |
| Propylparaben | - | <0.5 – 5.8 **(b)**  (River) | 43.0 – 1540 **(b)** | <0.5 – 28.0 **(b)**  (Secondary Treatment) |  |  | Switzerland | Jonkers *et al.*, 2009 |
| Pyrimethanil | - | 2.2 - 7.1 **(b)**  (River) |  |  |  |  | Cameroon | Branchet *et al.*, 2018 |
| Pyrimethanil | - | 47.6 / 162.8 **(e)**  (River) | 272.3 - 856.9 **(e)** | 191.1 / 643.0 **(e)** (Secondary Treatment)  146.5 / 574.2 **(e)**  (Tertiary Treatment) |  |  | Greece | Stamatis, Hela and Konstantinou, 2010 |
| Sertaconazole | - |  |  |  | 40.0 **(d)** |  | Spain | Castro *et al.*, 2016 |
| Sertaconazole | - |  |  |  | 11.0 – 76.0 **(b)** |  | Spain | Castro *et al.*, 2021 |
| Sertaconazole | - | 9.8 ± 1.0 **(a)** | 14.4 ± 0.4 **(a)** | ND |  |  | China | Wang *et al.*, 2018 |
| Sulfamethoxazole | - | 2.9 – 26.0 **(b)**  (River) |  |  |  |  | China | Peng *et al.*, 2018 |
| Sulfamethoxazole | - | 210.0 ± 29.0**(a)** | 217.0 **(c)** | 49.4 **(c)** |  |  | China | Yang *et al.*, 2017 |
| Sulfamethoxazole | 16000 |  | 9054.4 **(a)** |  | 3.3 |  | South Africa | Faleye *et al.*, 2019 |
| Sulfamethoxazole | - |  | 12.0 – 2204.0 **(b)** | 18.0 – 8263.0 **(b)** |  |  | Germany | Rossmann *et al.*, 2014 |
| Sulfamethoxazole | - | 965.0 **(a)**  (Groundwater) |  |  |  |  | USA | Elliott *et al.*, 2018 |
| Sulfamethoxazole | - | 332000.0 **(a)** |  | 5290000.0 **(a)** |  |  | Nigeria | Hu *et al.*, 2021 |
| Sulfamethoxazole | - | 27.9 / 78.9 **(e)**  (River) |  |  |  |  | China | Wang *et al.*, 2015 |
| Sulfamethoxazole | - |  | 220.0 **(d)**  (STP1)  100.0 **(d)**  (STP2) | 260.0 **(d)**  (STP1)  25.0 **(d)**  (STP2) | ND  (STP1)  <LOQ  (STP2) |  | India | Subedi *et al.*, 2017 |
| Sulfaphenazole | - | 58.0 – 149.0 **(b)**  (River) |  |  |  |  | Spain | Boleda, Galceran and Ventura, 2013 |
| Tebuconazole | - | 0.3 **/** 0.8 **(e)**  (Groundwater) |  |  |  |  | China | Peng *et al.*, 2014 |
| Tebuconazole | - |  |  | 21.0 - 86.0 **(b)**  (sequencing batch reactor (SBR) with activated sludge);  3.0 – 21.0 **(b)**  (pre-treatment, primary treatment, conventional activated sludge biological treatment and final decantation ) |  |  | Spain | Campos-Mañas *et al.*, 2019 |
| Tebuconazole | - | 3.0  (River) | <LOQ | 2.0 **(d)** | ND;  < MLQ  (Sewage) | ND | China | Huang *et al.*, 2010 |
| Tebuconazole | - | 9100.0 **(a)**  (River) |  |  |  |  | Germany | Berenzen *et al.*, 2005 |
| Tebuconazole | - | 1.8 - 2.7 **(b)**  (River) |  |  |  |  | Bangladesh | Angeles *et al.*, 2020 |
| Tebuconazole | - | 0.0  (River) |  |  |  |  | Cameroon | Branchet *et al.*, 2018 |
| Tebuconazole | - | 3.6 - 133.4 **(b)**  (River) |  |  |  |  | China | Peng *et al.*, 2018 |
| Tebuconazole | - | 1.7 - 15.4 **(b)**  (River) |  |  |  |  | Spain | Ccanccapa *et al.*, 2016 |
| Tebuconazole | - | 73.4 / 105.3 **(e)**  (River) | 564.1 / 1893.0 **(e)** | 337.7 / 1120.0 **(e)**  (Secondary Treatment)  232.9 / 691.1 **(e)**  (Tertiary Treatment) |  |  | Greece | Stamatis, Hela and Konstantinou, 2010 |
| Tebuconazole | - | 1.0 |  | 20.0 - 55.0 **(b)** |  |  | USA | Angeles *et al.*, 2021 |
| Tebuconazole | - |  |  |  | <LOQ – 13.6 ± 0.2 **(b)** |  | Spain | García‐Valcárcel and Tadeo, 2011 |
| Tebuconazole | - |  |  | 7.7  8.6  7.1  (Different WWTPs) |  |  | Canada | Westlund and Yargeau, 2017 |
| Tebuconazole | - |  | ND – 8.0 **(b)** | 1.0 – 10.0 **(b)** |  |  | Switzerland | Kahle *et al.*, 2008 |
| Tebuconazole | - | 2.4 ± 0.2  (River)  5.9 ± 1.2  (Stream 1)  11.0 ± 1  (Stream 2) | < LOQ  (WWTP 1)  8.9 ± 2.8  (WWTP 2) | 3.6 ± 0.3  (WWTP 1)  6.4 ± 1.6  (WWTP 2) | < LOQ *** |  | Germany | Wick, Fink and Ternes, 2010 |
| Tebuconazole | - | < LOQ  (River) |  |  |  |  | Spain | Bijlsma *et al.*, 2021 |
| Tebuconazole | - | ND – 4.2 **(b)**  (River) |  |  |  | 2.5 **(a)** | China | Huang *et al.*, 2013 |
| Tebuconazole | - | <0.1 – 447.0 **(b)**  (River) |  |  |  |  | Spain | Rico *et al.*, 2019 |
| Tebuconazole | - |  |  | 7.0 – 77.0 **(b)** |  |  | Canada | Elkayar *et al.*, 2022 |
| Tebuconazole | - | 252.4 ± 70.2 | 115.1 ± 37.6 | 927.5 ± 2.4 |  |  | England | Wattanayon and Kasprzyk-Hordern, 2021 |
| Terbinafine | - |  | < LOQ | < LOQ | 4.0 – 40.0 **(b)** (Dewatered Sludge) |  | Sweden | Lindberg, Fick and Tysklind, 2010 |
| Terbinafine | - | 4000.0 – 15000.0 **(b)**  (Lake)  98.0 - 1800.0 **(b)**  (Groundwater)  29.0 – 190.0 **(b)**  (River) |  | 120.0  (Activated Sludge Treatment) |  |  | India | Fick *et al.*, 2009 |
| Terbinafine | - | 50.2 ± 6.5 | ND | 30.5 ± 2.4 |  |  | England | Wattanayon and Kasprzyk-Hordern, 2021 |
| Terbinafine | - |  | < LOQ - 27.0 **(b)**  (aqueous + solid) | < LOQ - 5.0 **(b)** | < LOQ - 300.0 **(b)**  (Digested sludge) |  | Sweden | Östman *et al.*, 2017 |
| Terbinafine | - |  | 510.0 | 120.0 |  |  | USA | Bisceglia *et al.*, 2010 |
| Terbinafine | - | 130.0 **(d)**  (River)  540.0 **(d)**  (Aquifers) |  |  |  |  | India | Velpandian *et al.*, 2018 |
| Terbinafine | - |  | 3.9 **(a)** | 1.0 **(a)** |  |  | Sweden | Östman *et al.*, 2019 |
| Terbuthylazine | - |  |  |  | 4.1  (Sewage) |  | Greece | Miserli, Nastopoulou and Konstantinou, 2022 |
| Terbuthylazine | - | 2.4 ± 0.1  (River)  13.0 ± 1  (Stream 1)  2.9 ± 0.3  (Stream 2) | < LOQ  (WWTP 1)  18.0 ± 2  (WWTP 2) | < LOQ  (WWTP 1)  33.0 ± 1  (WWTP 2) | < LOQ *** |  | Germany | Wick, Fink and Ternes, 2010 |
| Tetraconazole | - | 1.7 - 8.9 **(b)**  (River) |  |  |  |  | Cameroon | Branchet *et al.*, 2018 |
| Thiabendazole | - |  |  |  | 11.4 – 186.0 **(b)**  (Dewatered) | 0.29 **(b)**  (Soil) | Spain | Masiá *et al.*, 2015 |
| Thiabendazole | - | 125.0 **(a)**  (Drinking) |  |  |  |  | Canada | Husk *et al.*, 2019 |
| Thiabendazole | - |  | 0.9 **(c)** | 1.0 **(c)** |  |  | China | Yang *et al.*, 2017 |
| Thiabendazole | - |  |  | 341.0 - 926.0 **(b)**  (sequencing batch reactor (SBR) with activated sludge) |  |  | Spain | Campos-Mañas *et al.*, 2019 |
| Thiabendazole | - |  |  |  | < LOQ - 7.2 ± 0.7 **(b)** |  | Spain | García‐Valcárcel and Tadeo, 2011 |
| Thiabendazole | - |  |  |  | 5.0 – 13.0 **(b)** |  | Spain | Castro *et al.*, 2021 |
| Thiabendazole | - | 34500.0 **(a)**  (River) |  |  |  |  | Spain | Bijlsma *et al.*, 2021 |
| Thiabendazole | - |  | 6.8 – 220.0 **(b)** | 6.2 – 100.0 **(b)** |  |  | Canada | Guerra *et al.*, 2014 |
| Thiabendazole | - |  |  |  | 4.4 ± 0.13 **(a)**  (sewage) |  | Brazil | Cerqueira *et al.*, 2018 |
| Thiabendazole | - |  |  |  | <LOQ - 17.5 **(b)**  (mixed WWTP) |  | South Korea | Subedi *et al.*, 2014 |
| Thiabendazole | - | 37.0 – 75.0 **(b)**  (River) |  |  |  |  | Costa Rica | Ramírez-Morales *et al.*, 2021 |
| Thiabendazole | - | 330.0 – 12250.0 **(b)**  (River) |  | 160.0 – 1770.0 **(b)** |  |  | Mexico | Salvatierra-stamp *et al.*, 2018 |
| Thiabendazole | - | 5.0 **(a)**  (River) |  |  |  |  | Romania | Chițescu and Nicolau, 2014 |
| Thiabendazole | - | 53.0 **(a)**  (River) |  |  |  |  | Romania | Chitescu *et al.*, 2015 |
| Thiabendazole | - | 0.4 – 48.8 **(b)**  (River) |  |  |  |  | Spain | Ccanccapa *et al.*, 2016 |
| Thiabendazole | - |  | 1570000.0 **(a)** | 120000.0 **(a)** |  |  | Spain | Peris‐Vicente *et al.*, 2016 |
| Thiabendazole | - | 10.0 **(a)**  (Surface) |  |  |  |  | The Netherlands | Chitescu *et al.*, 2012 |
| Thiabendazole | - | 37.3  (Surface)  ND  (Groundwater) |  |  |  |  | USA | Bradley *et al.*, 2016 |
| Thiabendazole | - |  |  |  | 3033.2  (solid fase) |  | Spain | Rivas Ibáñez *et al.*, 2015 |
| Thiabendazole | - |  | 0.13 - 0.68 **(b)** * | 0.10 - 0.83 **(b)** * | 0.01 - 0.03 **(b)** * |  | China | Liu *et al.*, 2017 |
| Thiabendazole | - | 130.0 | 18.0 - 730.0 **(b)** |  |  |  | China and Sweden | Angeles *et al.*, 2021 |
| Thiabendazole | - |  | 1900000.0 **(a)** | 120000.0 **(a)** | 410000.0 **(a)** |  | Spain | Romero-Cano *et al.*, 2015 |
| Thiabendazole | - |  |  | 0.4 ± 0.1  (After Treatment) |  |  | China | Chen *et al.*, 2013 |
| Thiabendazole | - | 0.7 ± 0.1  (River)  18.0 ± 3  (Stream 1)  5.4 ± 2.0  (Stream 2) | < LOQ  (WWTP 1)  13.0 ± 2  (WWTP 2) | 4.7 ± 0.6  (WWTP 1)  13.0 ± 1  (WWTP 2) | 6.7 ± 4.0 *** |  | Germany | Wick, Fink and Ternes, 2010 |
| Thiabendazole | - | ND  (River Reservoir) | 0.4 ± 0.1 **(d)** | 0.4 ± 0.1 **(d)** | 3.2 ± 0.1 **(d)**  (Dewatered) | ND | China | Chen *et al.*, 2012 |
| Thiabendazole | - | 8.03 ± 4.47 **(d)** | 7.1 ± 2.21 **(d)** | 6.2 ± 3.26 **(d)** | 0.006 **(d)** | 2.8 ± 1.61**(d)** | Thailand | Juksu *et al.*, 2019 |
| Thiabendazole | - |  | 64.0 **(d)**  (STP1)  123.0 **(d)**  (STP2) | 79.0 **(d)**  (STP1)  25.0 **(d)**  (STP2) | ND  (STP1)  3.5 **(d)**  (STP2) |  | India | Subedi *et al.*, 2017 |
| Tioconazole | - |  |  |  | 11.0 – 378.0 **(b)** |  | Spain | Castro *et al.*, 2021 |
| Tioconazole | - |  |  |  | 74.0 **(d)** |  | Spain | Castro *et al.*, 2016 |
| Tinidazole | - | 0.0 **(d)**  (River)  10.0 **(d)**  (Aquifers) |  |  |  |  | India | Velpandian *et al.*, 2018 |
| Triadimefon | - | < LOD  (River) | 24.2 / 115.0 **(e)** | 7.7 / 28.8 **(e)**  (Secondary Treatment)  1.5 / 10.4 **(e)**  (Tertiary Treatment) |  |  | Greece | Stamatis, Hela and Konstantinou, 2010 |
| Triclocarban | - |  | 14.0 – 270.0 **(b)** | 3.1 – 33.0 **(b)** |  |  | Canada | Guerra *et al.*, 2014 |
| Triclocarban | 661.0 | 2.07 **(d)**  (Xizhijiang River)  32.8 **(d)**  (Shima River) |  |  |  | 109.0 **(d)**  (Xizhijiang River)  334.0 **(d)**  (Shima River) | China | Chen *et al.*, 2014 |
| Tricyclazole | - | 0.4 - 7.8 **(b)**  (River, Lake and Canal) |  |  |  |  | Bangladesh | Angeles *et al.*, 2020 |
| Tricyclazole | - | 18.4 - 149.9 **(b)**  (Canal) |  |  |  |  | China | Cheng *et al.*, 2023 |
| Triticonazole | 2.5  (River) |  |  |  |  |  | Korea | Lee *et al.*, 2024 |
| Voriconazole | - |  | < LOQ  (Hospital) | < LOQ  (Hospital) |  |  | Germany | Knoth *et al.*, 2018 |
| Voriconazole | - | 2500000.0 **(a)**  (Rivers and Lakes) |  |  |  |  | India | Lübbert *et al.*, 2017 |
| Voriconazole | - | 0.0 **(d)**  (River)  90.0 **(d)**  (Aquifers) |  |  |  |  | India | Velpandian *et al.*, 2018 |
| Voriconazole | - | ND | ND | ND |  |  | England | Wattanayon and Kasprzyk-Hordern, 2021 |
| Voriconazole | - |  | <LOQ | <LOQ |  |  | Germany | Gurke *et al.*, 2015 |

**Caption:** All values are rounded to one decimal point. **LOD** - Limit of Detection; **LOQ** - Limit of Quantification; **MQL –** Method Quantification Limit; **ND** - Not Detected; **PNEC** - Predicted No Effect Concentration; **STP** - Sewage Treatment Plant; **WW** – Wastewater; **WWTP** - Wastewater Treatment Plant; **(a)** maximum; **(b)** min-max; **(c)** median; **(d)** mean; **(e)** mean/max; * (µg/d/person); ** (mg/person/d); *** (ng g TSS^-1^)

**References used**

Abou-Elwafa Abdallah, M. *et al.* (2019) “A single run, rapid polarity switching method for determination of 30 pharmaceuticals and personal care products in waste water using Q-Exactive Orbitrap high resolution accurate mass spectrometry,” *Journal of Chromatography A*, 1588, pp. 68–76. Available at: https://doi.org/10.1016/j.chroma.2018.12.033.

Angeles, L.F. *et al.* (2020) “Retrospective suspect screening reveals previously ignored antibiotics, antifungal compounds, and metabolites in Bangladesh surface waters,” *Science of The Total Environment*, 712, p. 136285. Available at: https://doi.org/10.1016/j.scitotenv.2019.136285.

Angeles, L.F. *et al.* (2021) “Increased coverage and high confidence in suspect screening of emerging contaminants in global environmental samples,” *Journal of Hazardous Materials*, 414, p. 125369. Available at: https://doi.org/10.1016/j.jhazmat.2021.125369.

Asghar, M.A. *et al.* (2018) “Suspect screening and target quantification of human pharmaceutical residues in the surface water of Wuhan, China, using UHPLC-Q-Orbitrap HRMS,” *Science of The Total Environment*, 635, pp. 828–837. Available at: https://doi.org/10.1016/j.scitotenv.2018.04.179.

Assress, H.A. *et al.* (2019) “Target quantification of azole antifungals and retrospective screening of other emerging pollutants in wastewater effluent using UHPLC –QTOF-MS,” *Environmental Pollution*, 253, pp. 655–666. Available at: https://doi.org/10.1016/j.envpol.2019.07.075.

Assress, H.A. *et al.* (2020) “Occurrence and risk assessment of azole antifungal drugs in water and wastewater,” *Ecotoxicology and Environmental Safety*, 187, p. 109868. Available at: https://doi.org/10.1016/j.ecoenv.2019.109868.

de Barros, A.L.C. *et al.* (2018) “Determination of nine pharmaceutical active compounds in surface waters from Paraopeba River Basin in Brazil by LTPE-HPLC-ESI-MS/MS,” *Environmental Science and Pollution Research*, 25(20), pp. 19962–19974. Available at: https://doi.org/10.1007/s11356-018-2123-y.

Berenzen, N. *et al.* (2005) “A comparison of predicted and measured levels of runoff-related pesticide concentrations in small lowland streams on a landscape level,” *Chemosphere*, 58(5), pp. 683–691. Available at: https://doi.org/10.1016/j.chemosphere.2004.05.009.

Bijlsma, L. *et al.* (2021) “Ecological risk assessment of pesticides in the Mijares River (eastern Spain) impacted by citrus production using wide-scope screening and target quantitative analysis,” *Journal of Hazardous Materials*, 412, p. 125277. Available at: https://doi.org/10.1016/j.jhazmat.2021.125277.

Bisceglia, K.J. *et al.* (2010) “Trace determination of pharmaceuticals and other wastewater-derived micropollutants by solid phase extraction and gas chromatography/mass spectrometry,” *Journal of Chromatography A*, 1217(4), pp. 558–564. Available at: https://doi.org/10.1016/j.chroma.2009.11.062.

Boleda, M.R., Galceran, M.T. and Ventura, F. (2013) “Validation and uncertainty estimation of a multiresidue method for pharmaceuticals in surface and treated waters by liquid chromatography–tandem mass spectrometry,” *Journal of Chromatography A*, 1286, pp. 146–158. Available at: https://doi.org/10.1016/j.chroma.2013.02.077.

Bradley, P.M. *et al.* (2016) “Pre/post-closure assessment of groundwater pharmaceutical fate in a wastewater-facility-impacted stream reach,” *Science of The Total Environment*, 568, pp. 916–925. Available at: https://doi.org/10.1016/j.scitotenv.2016.06.104.

Branchet, P. *et al.* (2018) “Polar pesticide contamination of an urban and peri-urban tropical watershed affected by agricultural activities (Yaoundé, Center Region, Cameroon),” *Environmental Science and Pollution Research*, 25(18), pp. 17690–17715. Available at: https://doi.org/10.1007/s11356-018-1798-4.

Burzio, C. *et al.* (2022) “Removal of organic micropollutants from municipal wastewater by aerobic granular sludge and conventional activated sludge,” *Journal of Hazardous Materials*, 438, p. 129528. Available at: https://doi.org/10.1016/j.jhazmat.2022.129528.

Campos-Mañas, M.C. *et al.* (2019) “Determination of pesticide levels in wastewater from an agro-food industry: Target, suspect and transformation product analysis.,” *Chemosphere*, 232, pp. 152–163. Available at: https://doi.org/10.1016/j.chemosphere.2019.05.147.

Cangola, J., Abagale, F.K. and Cobbina, S.J. (2024) “A systematic review of pharmaceutical and personal care products as emerging contaminants in waters: The panorama of West Africa,” *Science of The Total Environment*, 911, p. 168633. Available at: https://doi.org/10.1016/j.scitotenv.2023.168633.

Casado, J. *et al.* (2014) “Selective determination of antimycotic drugs in environmental water samples by mixed-mode solid-phase extraction and liquid chromatography quadrupole time-of-flight mass spectrometry,” *Journal of Chromatography A*, 1339, pp. 42–49. Available at: https://doi.org/10.1016/j.chroma.2014.02.087.

Casado, J. *et al.* (2015) “Selective extraction of antimycotic drugs from sludge samples using matrix solid-phase dispersion followed by on-line clean-up,” *Analytical and Bioanalytical Chemistry*, 407(3), pp. 907–917. Available at: https://doi.org/10.1007/s00216-014-8167-z.

Castro, G. *et al.* (2016) “Identification and determination of chlorinated azoles in sludge using liquid chromatography quadrupole time-of-flight and triple quadrupole mass spectrometry platforms,” *Journal of Chromatography A*, 1476, pp. 69–76. Available at: https://doi.org/10.1016/j.chroma.2016.11.020.

Castro, G. *et al.* (2021) “Identification and determination of emerging pollutants in sewage sludge driven by UPLC-QTOF-MS data mining,” *Science of The Total Environment*, 778, p. 146256. Available at: https://doi.org/10.1016/j.scitotenv.2021.146256.

Ccanccapa, A. *et al.* (2016) “Pesticides in the Ebro River basin: Occurrence and risk assessment,” *Environmental Pollution*, 211, pp. 414–424. Available at: https://doi.org/10.1016/j.envpol.2015.12.059.

Cerqueira, M.B.R. *et al.* (2018) “Sample as solid support in MSPD: A new possibility for determination of pharmaceuticals, personal care and degradation products in sewage sludge,” *Chemosphere*, 211, pp. 875–883. Available at: https://doi.org/10.1016/j.chemosphere.2018.07.165.

Chen, Z.-F. *et al.* (2012) “Determination of biocides in different environmental matrices by use of ultra-high-performance liquid chromatography–tandem mass spectrometry,” *Analytical and Bioanalytical Chemistry*, 404(10), pp. 3175–3188. Available at: https://doi.org/10.1007/s00216-012-6444-2.

Chen, Z.-F. *et al.* (2013) “Occurrence and dissipation of three azole biocides climbazole, clotrimazole and miconazole in biosolid-amended soils,” *Science of The Total Environment*, 452–453, pp. 377–383. Available at: https://doi.org/10.1016/j.scitotenv.2013.03.004.

Chen, Z.-F. *et al.* (2014) “Triclosan as a surrogate for household biocides: An investigation into biocides in aquatic environments of a highly urbanized region,” *Water Research*, 58, pp. 269–279. Available at: https://doi.org/10.1016/j.watres.2014.03.072.

Cheng, X. *et al.* (2023) “Analysis of Emerging Contaminants in Surface Water, Aquaculture Ponds and Wastewater Treatment Facilities in the Taige Canal Basin,” *Chemical Research in Chinese Universities*, 39(3), pp. 516–524. Available at: https://doi.org/10.1007/s40242-023-3073-6.

Chitescu, C.L. *et al.* (2012) “Accurate mass screening of pharmaceuticals and fungicides in water by U-HPLC–Exactive Orbitrap MS,” *Analytical and Bioanalytical Chemistry*, 403(10), pp. 2997–3011. Available at: https://doi.org/10.1007/s00216-012-5888-8.

Chitescu, C.L. *et al.* (2015) “High sensitive multiresidue analysis of pharmaceuticals and antifungals in surface water using U-HPLC-Q-Exactive Orbitrap HRMS. Application to the Danube river basin on the Romanian territory,” *Science of The Total Environment*, 532, pp. 501–511. Available at: https://doi.org/10.1016/j.scitotenv.2015.06.010.

Chițescu, C.L. and Nicolau, A.I. (2014) “Preliminary survey of pharmaceutical residues in some important Romanian rivers,” *Toxicological & Environmental Chemistry*, 96(9), pp. 1333–1345. Available at: https://doi.org/10.1080/02772248.2015.1005092.

Coors, A. *et al.* (2018) “Prospective environmental risk assessment of mixtures in wastewater treatment plant effluents – Theoretical considerations and experimental verification,” *Water Research*, 140, pp. 56–66. Available at: https://doi.org/10.1016/j.watres.2018.04.031.

Dehm, J. *et al.* (2021) “Screening of pharmaceuticals in coastal waters of the southern coast of Viti Levu in Fiji, South Pacific,” *Chemosphere*, 276, p. 130161. Available at: https://doi.org/10.1016/j.chemosphere.2021.130161.

Ebele, A.J. *et al.* (2020) “Occurrence, seasonal variation and human exposure to pharmaceuticals and personal care products in surface water, groundwater and drinking water in Lagos State, Nigeria,” *Emerging Contaminants*, 6, pp. 124–132. Available at: https://doi.org/10.1016/j.emcon.2020.02.004.

Elkayar, K. *et al.* (2022) “Passive sampling and in vitro assays to monitor antiandrogens in a river affected by wastewater discharge,” *Science of The Total Environment*, 804, p. 150067. Available at: https://doi.org/10.1016/j.scitotenv.2021.150067.

Elliott, S.M. *et al.* (2018) “Concentrations of pharmaceuticals and other micropollutants in groundwater downgradient from large on-site wastewater discharges,” *PLOS ONE*, 13(11), p. e0206004. Available at: https://doi.org/10.1371/journal.pone.0206004.

Escher, B.I. *et al.* (2011) “Environmental toxicology and risk assessment of pharmaceuticals from hospital wastewater,” *Water Research*, 45(1), pp. 75–92. Available at: https://doi.org/10.1016/j.watres.2010.08.019.

Fáberová, M. *et al.* (2017) “Frequency and use of pharmaceuticals in selected Slovakian town via wastewater analysis,” *Monatshefte für Chemie - Chemical Monthly*, 148(3), pp. 441–448. Available at: https://doi.org/10.1007/s00706-016-1853-0.

Faleye, A.C. *et al.* (2019) “Concentration and reduction of antibiotic residues in selected wastewater treatment plants and receiving waterbodies in Durban, South Africa,” *Science of The Total Environment*, 678, pp. 10–20. Available at: https://doi.org/10.1016/j.scitotenv.2019.04.410.

Fick, J. *et al.* (2009) “Contamination of surface, ground, and drinking water from pharmaceutical production,” *Environmental Toxicology and Chemistry*, 28(12), pp. 2522–2527. Available at: https://doi.org/10.1897/09-073.1.

Fick, J. and Lindberg, R.H. (2014) *Analysis of pharmaceuticals and hormones in samples from WWTPs and receiving waters*. Available at: https://www.diva-portal.org/smash/get/diva2:927576/FULLTEXT01.pdf.

Folarin, O.S. *et al.* (2020) “Occurrence of Acetaminophen, Amoxicillin, Diclofenac and Methylparaben in Lagos and Ologe Lagoons, Lagos, Nigeria,” *Journal of Applied Sciences and Environmental Management*, 23(12), p. 2143. Available at: https://doi.org/10.4314/jasem.v23i12.10.

Frédéric, O. and Yves, P. (2014) “Pharmaceuticals in hospital wastewater: Their ecotoxicity and contribution to the environmental hazard of the effluent,” *Chemosphere*, 115, pp. 31–39. Available at: https://doi.org/10.1016/j.chemosphere.2014.01.016.

García‐Valcárcel, A.I. and Tadeo, J.L. (2011) “Determination of azoles in sewage sludge from Spanish wastewater treatment plants by liquid chromatography‐tandem mass spectrometry,” *Journal of Separation Science*, 34(11), pp. 1228–1235. Available at: https://doi.org/10.1002/jssc.201000814.

Grabicova, K. *et al.* (2015) “Presence of pharmaceuticals in benthic fauna living in a small stream affected by effluent from a municipal sewage treatment plant,” *Water Research*, 72, pp. 145–153. Available at: https://doi.org/10.1016/j.watres.2014.09.018.

Guerra, P. *et al.* (2014) “Occurrence and fate of antibiotic, analgesic/anti-inflammatory, and antifungal compounds in five wastewater treatment processes,” *Science of The Total Environment*, 473–474, pp. 235–243. Available at: https://doi.org/10.1016/j.scitotenv.2013.12.008.

Gurke, R. *et al.* (2015) “Development of a SPE-HPLC–MS/MS method for the determination of most prescribed pharmaceuticals and related metabolites in urban sewage samples,” *Journal of Chromatography B*, 990, pp. 23–30. Available at: https://doi.org/10.1016/j.jchromb.2015.03.008.

Hanafiah, Z.M. *et al.* (2024) “Pharmaceutical Footprint in Domestic Wastewater: Case Study in Malaysia,” *Water, Air, & Soil Pollution*, 235(1), p. 51. Available at: https://doi.org/10.1007/s11270-023-06844-1.

Helwig, K. *et al.* (2015) “Ranking prescribed pharmaceuticals in terms of environmental risk: Inclusion of hospital data and the importance of regular review,” *Environmental Toxicology and Chemistry*, 35(4), pp. 1043–1050. Available at: https://doi.org/10.1002/etc.3302.

Holton, E. *et al.* (2022) “Spatiotemporal urban water profiling for the assessment of environmental and public exposure to antimicrobials (antibiotics, antifungals, and antivirals) in the Eerste River Catchment, South Africa,” *Environment International*, 164, p. 107227. Available at: https://doi.org/10.1016/j.envint.2022.107227.

Hossain, A. *et al.* (2018) “Occurrence and ecological risk of pharmaceuticals in river surface water of Bangladesh,” *Environmental Research*, 165, pp. 258–266. Available at: https://doi.org/10.1016/j.envres.2018.04.030.

Hu, L.-X. *et al.* (2021) “What is in Nigerian waters? Target and non-target screening analysis for organic chemicals,” *Chemosphere*, 284, p. 131546. Available at: https://doi.org/10.1016/j.chemosphere.2021.131546.

Huang, Q. *et al.* (2010) “Determination of commonly used azole antifungals in various waters and sewage sludge using ultra-high performance liquid chromatography–tandem mass spectrometry,” *Journal of Chromatography A*, 1217(21), pp. 3481–3488. Available at: https://doi.org/10.1016/j.chroma.2010.03.022.

Huang, Q. *et al.* (2012) “Enantiomeric determination of azole antifungals in wastewater and sludge by liquid chromatography–tandem mass spectrometry,” *Analytical and Bioanalytical Chemistry*, 403(6), pp. 1751–1760. Available at: https://doi.org/10.1007/s00216-012-5976-9.

Huang, Q. *et al.* (2013) “Chiral profiling of azole antifungals in municipal wastewater and recipient rivers of the Pearl River Delta, China,” *Environmental Science and Pollution Research*, 20(12), pp. 8890–8899. Available at: https://doi.org/10.1007/s11356-013-1862-z.

Husk, B. *et al.* (2019) “Pharmaceuticals and pesticides in rural community drinking waters of Quebec, Canada – a regional study on the susceptibility to source contamination,” *Water Quality Research Journal*, 54(2), pp. 88–103. Available at: https://doi.org/10.2166/wqrj.2019.038.

Jonkers, N. *et al.* (2009) “Mass flows of endocrine disruptors in the Glatt River during varying weather conditions,” *Environmental Pollution*, 157(3), pp. 714–723. Available at: https://doi.org/10.1016/j.envpol.2008.11.029.

Juksu, K. *et al.* (2019) “Occurrence, fate and risk assessment of biocides in wastewater treatment plants and aquatic environments in Thailand,” *Science of The Total Environment*, 690, pp. 1110–1119. Available at: https://doi.org/10.1016/j.scitotenv.2019.07.097.

Kahle, M. *et al.* (2008) “Azole Fungicides: Occurrence and Fate in Wastewater and Surface Waters,” *Environmental Science & Technology*, 42(19), pp. 7193–7200. Available at: https://doi.org/10.1021/es8009309.

Kårelid, V., Larsson, G. and Björlenius, B. (2017a) “Effects of recirculation in a three-tank pilot-scale system for pharmaceutical removal with powdered activated carbon,” *Journal of Environmental Management*, 193, pp. 163–171. Available at: https://doi.org/10.1016/j.jenvman.2017.01.078.

Kårelid, V., Larsson, G. and Björlenius, B. (2017b) “Pilot-scale removal of pharmaceuticals in municipal wastewater: Comparison of granular and powdered activated carbon treatment at three wastewater treatment plants,” *Journal of Environmental Management*, 193, pp. 491–502. Available at: https://doi.org/10.1016/j.jenvman.2017.02.042.

Karthikraj, R. and Kannan, K. (2017) “Mass loading and removal of benzotriazoles, benzothiazoles, benzophenones, and bisphenols in Indian sewage treatment plants,” *Chemosphere*, 181, pp. 216–223. Available at: https://doi.org/10.1016/j.chemosphere.2017.04.075.

Kim, J.-W. *et al.* (2009) “Acute toxicity of pharmaceutical and personal care products on freshwater crustacean (Thamnocephalus platyurus) and fish (Oryzias latipes),” *The Journal of Toxicological Sciences*, 34(2), pp. 227–232. Available at: https://doi.org/10.2131/jts.34.227.

Knoth, H. *et al.* (2018) “Determination of the contamination by azole antimycotics in hospital and house sewage - A pilot project for the city of Dresden,” *Pharmazie*, 73(5), pp. 260–263. Available at: https://doi.org/https://doi.org/10.1691/ph.2018.8364.

Kotowska, U. and Bieńczyk, K. (2013) “Use of direct immersion solid-phase microextraction on polyacrylate and polydimethylsiloxane stationary phases for simultaneous determination of the neutral and basic pharmaceuticals in wastewater,” *Open Chemistry*, 11(10), pp. 1634–1643. Available at: https://doi.org/10.2478/s11532-013-0296-2.

Kuroda, K. *et al.* (2015) “Pepper mild mottle virus as an indicator and a tracer of fecal pollution in water environments: Comparative evaluation with wastewater-tracer pharmaceuticals in Hanoi, Vietnam,” *Science of The Total Environment*, 506–507, pp. 287–298. Available at: https://doi.org/10.1016/j.scitotenv.2014.11.021.

Lacey, C. *et al.* (2012) “Occurrence of pharmaceutical compounds in wastewater process streams in Dublin, Ireland,” *Environmental Monitoring and Assessment*, 184(2), pp. 1049–1062. Available at: https://doi.org/10.1007/s10661-011-2020-z.

Lee, S. *et al.* (2024) “Proposal for priority emerging pollutants in the Nakdong river, Korea: Application of EU watch list mechanisms,” *Environmental Pollution*, 341, p. 122838. Available at: https://doi.org/10.1016/j.envpol.2023.122838.

Li, W.-L. *et al.* (2018) “An evaluation on the intra-day dynamics, seasonal variations and removal of selected pharmaceuticals and personal care products from urban wastewater treatment plants,” *Science of The Total Environment*, 640–641, pp. 1139–1147. Available at: https://doi.org/10.1016/j.scitotenv.2018.05.362.

Lin, Y.-C. *et al.* (2015) “Occurrence of pharmaceuticals, hormones, and perfluorinated compounds in groundwater in Taiwan,” *Environmental Monitoring and Assessment*, 187(5), p. 256. Available at: https://doi.org/10.1007/s10661-015-4497-3.

Lindberg, R.H., Fick, J. and Tysklind, M. (2010) “Screening of antimycotics in Swedish sewage treatment plants – Waters and sludge,” *Water Research*, 44(2), pp. 649–657. Available at: https://doi.org/10.1016/j.watres.2009.10.034.

Liu, W.-R. *et al.* (2017) “Biocides in wastewater treatment plants: Mass balance analysis and pollution load estimation,” *Journal of Hazardous Materials*, 329, pp. 310–320. Available at: https://doi.org/10.1016/j.jhazmat.2017.01.057.

Loos, R. *et al.* (2013) “EU-wide monitoring survey on emerging polar organic contaminants in wastewater treatment plant effluents,” *Water Research*, 47(17), pp. 6475–6487. Available at: https://doi.org/10.1016/j.watres.2013.08.024.

López-Doval, J.C. *et al.* (2017) “Nutrients, emerging pollutants and pesticides in a tropical urban reservoir: Spatial distributions and risk assessment,” *Science of The Total Environment*, 575, pp. 1307–1324. Available at: https://doi.org/10.1016/j.scitotenv.2016.09.210.

Lopez-Herguedas, N. *et al.* (2022) “Characterization of the contamination fingerprint of wastewater treatment plant effluents in the Henares River Basin (central Spain) based on target and suspect screening analysis,” *Science of The Total Environment*, 806, p. 151262. Available at: https://doi.org/10.1016/j.scitotenv.2021.151262.

Lübbert, C. *et al.* (2017) “Environmental pollution with antimicrobial agents from bulk drug manufacturing industries in Hyderabad, South India, is associated with dissemination of extended-spectrum beta-lactamase and carbapenemase-producing pathogens,” *Infection*, 45(4), pp. 479–491. Available at: https://doi.org/10.1007/s15010-017-1007-2.

Macikova, P. *et al.* (2014) “Endocrine Disrupting Compounds Affecting Corticosteroid Signaling Pathways in Czech and Swiss Waters: Potential Impact on Fish,” *Environmental Science & Technology*, 48(21), pp. 12902–12911. Available at: https://doi.org/10.1021/es502711c.

Maraqa, M.A., Meetani, M. and Alhalabi, A.M. (2020) “Effectiveness of conventional wastewater treatment processes in removing pharmaceutically active compounds,” *IOP Conference Series: Earth and Environmental Science*, 424(1), p. 012014. Available at: https://doi.org/10.1088/1755-1315/424/1/012014.

Maruya, K.A. *et al.* (2015) “Which coastal and marine environmental contaminants are truly emerging?,” *Environmental Science and Pollution Research*, 22(3), pp. 1644–1652. Available at: https://doi.org/10.1007/s11356-014-2856-1.

Masiá, A. *et al.* (2015) “Assessment of two extraction methods to determine pesticides in soils, sediments and sludges. Application to the Túria River Basin,” *Journal of Chromatography A*, 1378, pp. 19–31. Available at: https://doi.org/10.1016/j.chroma.2014.11.079.

Miserli, K., Nastopoulou, A. and Konstantinou, I. (2022) “Removal of organic pollutants (pharmaceuticals and pesticides) from sewage sludge by hydrothermal carbonization using response surface methodology ( <scp>RSM</scp> ),” *Journal of Chemical Technology & Biotechnology*, 97(11), pp. 3111–3120. Available at: https://doi.org/10.1002/jctb.7178.

Monapathi, M. *et al.* (2021) “Antifungal agents, yeast abundance and diversity in surface water: Potential risks to water users,” *Chemosphere*, 274, p. 129718. Available at: https://doi.org/10.1016/j.chemosphere.2021.129718.

Montagna, M.T. *et al.* (2020) “Microbiological and Chemical Assessment of Wastewater Discharged by Infiltration Trenches in Fractured and Karstified Limestone (SCA.Re.S. Project 2019–2020),” *Pathogens*, 9(12), p. 1010. Available at: https://doi.org/10.3390/pathogens9121010.

Ngo, T.H. *et al.* (2021) “Occurrence of pharmaceutical and personal care products in Cau River, Vietnam,” *Environmental Science and Pollution Research*, 28(10), pp. 12082–12091. Available at: https://doi.org/10.1007/s11356-020-09195-0.

Östman, M. *et al.* (2017) “Screening of biocides, metals and antibiotics in Swedish sewage sludge and wastewater,” *Water Research*, 115, pp. 318–328. Available at: https://doi.org/10.1016/j.watres.2017.03.011.

Östman, M. *et al.* (2019) “Effect of full-scale ozonation and pilot-scale granular activated carbon on the removal of biocides, antimycotics and antibiotics in a sewage treatment plant,” *Science of The Total Environment*, 649, pp. 1117–1123. Available at: https://doi.org/10.1016/j.scitotenv.2018.08.382.

Peng, X. *et al.* (2012) “Distribution, behavior and fate of azole antifungals during mechanical, biological, and chemical treatments in sewage treatment plants in China,” *Science of The Total Environment*, 426, pp. 311–317. Available at: https://doi.org/10.1016/j.scitotenv.2012.03.067.

Peng, X. *et al.* (2014) “Occurrence and ecological potential of pharmaceuticals and personal care products in groundwater and reservoirs in the vicinity of municipal landfills in China,” *Science of The Total Environment*, 490, pp. 889–898. Available at: https://doi.org/10.1016/j.scitotenv.2014.05.068.

Peng, Y. *et al.* (2018) “Screening hundreds of emerging organic pollutants (EOPs) in surface water from the Yangtze River Delta (YRD): Occurrence, distribution, ecological risk,” *Environmental Pollution*, 241, pp. 484–493. Available at: https://doi.org/10.1016/j.envpol.2018.05.061.

Peris‐Vicente, J. *et al.* (2016) “Development and validation of a method to determine thiabendazole and o‐phenylphenol in wastewater using micellar liquid chromatography‐fluorescence detection,” *ELECTROPHORESIS*, 37(19), pp. 2517–2521. Available at: https://doi.org/10.1002/elps.201500580.

Peschka, M., Roberts, P.H. and Knepper, T.P. (2007) “Analysis, fate studies and monitoring of the antifungal agent clotrimazole in the aquatic environment,” *Analytical and Bioanalytical Chemistry*, 389(3), pp. 959–968. Available at: https://doi.org/10.1007/s00216-007-1480-z.

Qi, W. *et al.* (2015) “Elimination of polar micropollutants and anthropogenic markers by wastewater treatment in Beijing, China,” *Chemosphere*, 119, pp. 1054–1061. Available at: https://doi.org/10.1016/j.chemosphere.2014.09.027.

Ramírez-Morales, D. *et al.* (2021) “Pesticide occurrence and water quality assessment from an agriculturally influenced Latin-American tropical region,” *Chemosphere*, 262, p. 127851. Available at: https://doi.org/10.1016/j.chemosphere.2020.127851.

Ravichandran, M.K., Yoganathan, S. and Philip, L. (2021) “Removal and risk assessment of pharmaceuticals and personal care products in a decentralized greywater treatment system serving an Indian rural community,” *Journal of Environmental Chemical Engineering*, 9(6), p. 106832. Available at: https://doi.org/10.1016/j.jece.2021.106832.

Reis, E.O. *et al.* (2019) “Occurrence, removal and seasonal variation of pharmaceuticals in Brasilian drinking water treatment plants,” *Environmental Pollution*, 250, pp. 773–781. Available at: https://doi.org/10.1016/j.envpol.2019.04.102.

Rico, A. *et al.* (2019) “Identification of contaminants of concern in the upper Tagus river basin (central Spain). Part 1: Screening, quantitative analysis and comparison of sampling methods,” *Science of The Total Environment*, 666, pp. 1058–1070. Available at: https://doi.org/10.1016/j.scitotenv.2019.02.250.

Rivas Ibáñez, G. *et al.* (2015) “Fate of micropollutants during sewage sludge disintegration by low-frequency ultrasound,” *Chemical Engineering Journal*, 280, pp. 575–587. Available at: https://doi.org/10.1016/j.cej.2015.06.010.

Romero-Cano, R. *et al.* (2015) “Analysis of thiabendazole, 4-tert-octylphenol and chlorpyrifos in waste and sewage water by direct injection – micellar liquid chromatography,” *The Analyst*, 140(5), pp. 1739–1746. Available at: https://doi.org/10.1039/C4AN01782J.

Rossmann, J. *et al.* (2014) “Simultaneous determination of most prescribed antibiotics in multiple urban wastewater by SPE-LC–MS/MS,” *Journal of Chromatography B*, 969, pp. 162–170. Available at: https://doi.org/10.1016/j.jchromb.2014.08.008.

Sabater-Liesa, L. *et al.* (2021) “Retrospective mass spectrometric analysis of wastewater-fed mesocosms to assess the degradation of drugs and their human metabolites,” *Journal of Hazardous Materials*, 408, p. 124984. Available at: https://doi.org/10.1016/j.jhazmat.2020.124984.

Salvatierra-stamp, V. *et al.* (2018) “Hollow fiber liquid phase microextraction combined with liquid chromatography-tandem mass spectrometry for the analysis of emerging contaminants in water samples,” *Microchemical Journal*, 140, pp. 87–95. Available at: https://doi.org/10.1016/j.microc.2018.04.012.

Santos, L.H.M.L.M. *et al.* (2013) “Contribution of hospital effluents to the load of pharmaceuticals in urban wastewaters: Identification of ecologically relevant pharmaceuticals,” *Science of The Total Environment*, 461–462, pp. 302–316. Available at: https://doi.org/10.1016/j.scitotenv.2013.04.077.

Shi, Y. *et al.* (2020) “Antibiotics in wastewater from multiple sources and surface water of the Yangtze River in Chongqing in China,” *Environmental Monitoring and Assessment*, 192(3), p. 159. Available at: https://doi.org/10.1007/s10661-020-8108-6.

Stamatis, N., Hela, D. and Konstantinou, I. (2010) “Occurrence and removal of fungicides in municipal sewage treatment plant,” *Journal of Hazardous Materials*, 175(1–3), pp. 829–835. Available at: https://doi.org/10.1016/j.jhazmat.2009.10.084.

Van De Steene, J.C. and Lambert, W.E. (2008) “Validation of a solid-phase extraction and liquid chromatography–electrospray tandem mass spectrometric method for the determination of nine basic pharmaceuticals in wastewater and surface water samples,” *Journal of Chromatography A*, 1182(2), pp. 153–160. Available at: https://doi.org/10.1016/j.chroma.2008.01.012.

Van De Steene, J.C., Stove, C.P. and Lambert, W.E. (2010) “A field study on 8 pharmaceuticals and 1 pesticide in Belgium: Removal rates in waste water treatment plants and occurrence in surface water,” *Science of The Total Environment*, 408(16), pp. 3448–3453. Available at: https://doi.org/10.1016/j.scitotenv.2010.04.037.

Subedi, B. *et al.* (2014) “Emission of artificial sweeteners, select pharmaceuticals, and personal care products through sewage sludge from wastewater treatment plants in Korea,” *Environment International*, 68, pp. 33–40. Available at: https://doi.org/10.1016/j.envint.2014.03.006.

Subedi, B. *et al.* (2015) “Mass loading and removal of pharmaceuticals and personal care products, including psychoactive and illicit drugs and artificial sweeteners, in five sewage treatment plants in India,” *Journal of Environmental Chemical Engineering*, 3(4), pp. 2882–2891. Available at: https://doi.org/10.1016/j.jece.2015.09.031.

Subedi, B. *et al.* (2017) “Mass loading and removal of pharmaceuticals and personal care products including psychoactives, antihypertensives, and antibiotics in two sewage treatment plants in southern India,” *Chemosphere*, 167, pp. 429–437. Available at: https://doi.org/10.1016/j.chemosphere.2016.10.026.

Velpandian, T. *et al.* (2018) “Un-segregated waste disposal: an alarming threat of antimicrobials in surface and ground water sources in Delhi,” *Environmental Science and Pollution Research*, 25(29), pp. 29518–29528. Available at: https://doi.org/10.1007/s11356-018-2927-9.

Verlicchi, P. *et al.* (2012) “Hospital effluent: Investigation of the concentrations and distribution of pharmaceuticals and environmental risk assessment,” *Science of The Total Environment*, 430, pp. 109–118. Available at: https://doi.org/10.1016/j.scitotenv.2012.04.055.

Wang, Z. *et al.* (2015) “Comprehensive evaluation of pharmaceuticals and personal care products (PPCPs) in typical highly urbanized regions across China,” *Environmental Pollution*, 204, pp. 223–232. Available at: https://doi.org/10.1016/j.envpol.2015.04.021.

Wang, Z. *et al.* (2018) “Experimental and molecular docking study on graphene/Fe3O4 composites as a sorbent for magnetic solid-phase extraction of seven imidazole antifungals in environmental water samples prior to LC-MS/MS for enantiomeric analysis,” *Microchemical Journal*, 140, pp. 222–231. Available at: https://doi.org/10.1016/j.microc.2018.04.027.

Wattanayon, R. and Kasprzyk-Hordern, B. (2021) “A multi-residue chiral liquid chromatography coupled with tandem mass spectrometry method for analysis of antifungal agents and their metabolites in aqueous environmental matrices,” *Analytical Methods*, 13(22), pp. 2466–2477. Available at: https://doi.org/10.1039/D1AY00556A.

Westlund, P. and Yargeau, V. (2017) “Investigation of the presence and endocrine activities of pesticides found in wastewater effluent using yeast-based bioassays,” *Science of The Total Environment*, 607–608, pp. 744–751. Available at: https://doi.org/10.1016/j.scitotenv.2017.07.032.

Wick, A., Fink, G. and Ternes, T.A. (2010) “Comparison of electrospray ionization and atmospheric pressure chemical ionization for multi-residue analysis of biocides, UV-filters and benzothiazoles in aqueous matrices and activated sludge by liquid chromatography–tandem mass spectrometry,” *Journal of Chromatography A*, 1217(14), pp. 2088–2103. Available at: https://doi.org/10.1016/j.chroma.2010.01.079.

Wluka, A.-K. *et al.* (2016) “Analytical method development for the determination of eight biocides in various environmental compartments and application for monitoring purposes,” *Environmental Science and Pollution Research*, 23(21), pp. 21894–21907. Available at: https://doi.org/10.1007/s11356-016-7296-7.

Wroński, M., Trawiński, J. and Skibiński, R. (2024) “Antifungal drugs in the aquatic environment: A review on sources, occurrence, toxicity, health effects, removal strategies and future challenges,” *Journal of Hazardous Materials*, 465, p. 133167. Available at: https://doi.org/10.1016/j.jhazmat.2023.133167.

Yang, Y. *et al.* (2022) “Antibiotics and antimycotics in waste water treatment plants: Concentrations, removal efficiency, spatial and temporal variations, prediction, and ecological risk assessment,” *Environmental Research*, 215, p. 114135. Available at: https://doi.org/10.1016/j.envres.2022.114135.

Yang, Y.-Y. *et al.* (2017) “Suitability of pharmaceuticals and personal care products (PPCPs) and artificial sweeteners (ASs) as wastewater indicators in the Pearl River Delta, South China,” *Science of The Total Environment*, 590–591, pp. 611–619. Available at: https://doi.org/10.1016/j.scitotenv.2017.03.001.

Yilmaz, G. *et al.* (2017) “Characterization and toxicity of hospital wastewaters in Turkey,” *Environmental Monitoring and Assessment*, 189(2), p. 55. Available at: https://doi.org/10.1007/s10661-016-5732-2.

Zgoła‐Grześkowiak, A. and Grześkowiak, T. (2013) “Application of dispersive liquid–liquid microextraction followed by HPLC–MS/MS for the trace determination of clotrimazole in environmental water samples,” *Journal of Separation Science*, 36(15), pp. 2514–2521. Available at: https://doi.org/10.1002/jssc.201300271.

Zhang, N.-S. *et al.* (2015) “Ecological risks of home and personal care products in the riverine environment of a rural region in South China without domestic wastewater treatment facilities,” *Ecotoxicology and Environmental Safety*, 122, pp. 417–425. Available at: https://doi.org/10.1016/j.ecoenv.2015.09.004.

Zhao, P. *et al.* (2018) “Simultaneous enantioselective determination of six pesticides in aqueous environmental samples by chiral liquid chromatography with tandem mass spectrometry,” *Journal of Separation Science*, 41(6), pp. 1287–1297. Available at: https://doi.org/10.1002/jssc.201701259.
